# Co-occurrence of sudden feeding behaviour deviations and welfare issue onsets in growing-finishing pigs

**DOI:** 10.1186/s40813-025-00456-3

**Published:** 2025-08-07

**Authors:** Jacinta D. Bus, Rudi M. de Mol, Laura E. Webb, Eddie A. M. Bokkers, Iris J. M. M. Boumans

**Affiliations:** 1 Animal Production Systems group, Wageningen University & Research, Wageningen, 6700AH The Netherlands; 2 Wageningen Livestock Research, Wageningen, 6708WD The Netherlands

**Keywords:** Feeding pattern, Health issues, Heat stress, Dynamic linear model, Kalman filter, Feeding strategies

## Abstract

**Background:**

Modern sensor technologies and algorithms have the potential to continuously monitor indicators of individual animal welfare, but in growing-finishing pigs the validity of such welfare monitoring remains low for unclear reasons. This study explored how sudden deviations in individual pig feeding behaviour, detected as alerts by a dynamic linear model with Kalman filter, relate to the onset of welfare issues.

**Results:**

Alerts frequencies varied across feeding behaviour components, with higher occurrences for feed intake, feeding duration, feeding rate and night intake (approximately 14.5% of days with an alert) than for feeding frequency and circadian rhythm strength (approximately 7.7% of days with an alert). Limited temporal overlap was observed between feeding components (mean co-occurrence: 18 ± 2%, range 8–47%). Alert prevalence was lower in the first month of the growing-finishing phase for all feeding components except frequency and night intake, which showed opposing patterns. Substantial inter-individual variation in alert prevalence was observed (range: 1.1 – 22.2% alert days). The co-occurrence of alerts and welfare issue onsets, i.e. the sensitivity, was poor and not better than expected by chance (health issues: 2.0 – 48.7%, heat stress: 6.4 – 23.2%). Highest sensitivities were obtained for combinations of coughing, ear tip damage, lameness, rectal prolapse, or tail damage with feed intake, feeding duration or night intake. Sensitivities dropped further when only positive (range: 0.0 – 30.8%) or negative (range: 1.0 – 33.3%) alerts were considered. Sensitivities beyond chance expectations were obtained for feed intake, feeding duration and/or night intake in lame or tail-bitten pigs with specific feeding strategies (range: 4.6 – 66.7%).

**Conclusions:**

These results suggest that sudden deviations in feeding behaviour co-occur poorly with onsets of health issues and heat stress, and that current achievements may be largely based on statistical probabilities. However, mild sensitivities were identified for certain health issues and feeding components, especially for pigs with specific feeding strategies. In addition, the results imply that different types of deviations exist, which differ in suddenness and persistence across the welfare issues’ duration. Considering the importance of individual feeding strategies and basal feeding behaviour, stimulating more consistent basal behaviour by improving pigs’ housing conditions may reveal new avenues for continuous welfare monitoring.

**Supplementary Information:**

The online version contains supplementary material available at 10.1186/s40813-025-00456-3.

## Background

Modern sensor technologies have the potential to continuously monitor indicators of individual animal welfare [1–3]. Such monitoring can be used for early warning systems on-farm, but also to collect detailed longitudinal data that can help improve farm animal welfare retrospectively through changes in management and breeding. Welfare can be conceptualised as the balance between positive and negative experiences [4–6], though recent technological advancements have mostly focused on identifying negative experiences that impair animal welfare. Through continuous measurement of animal behaviour, it is possible to establish individual baselines from which deviations indicative of welfare issues can be isolated. Such behaviour-based approaches have so far been applied to detect issues like respiratory disease [7, 8], lameness [9–11] and heat stress [12, 13] in a range of farm animals.

In growing-finishing pigs, however, attempts to identify welfare issues through detection of deviations have not proven sufficiently valid, despite well-documented relationships between pig behaviour and welfare issues [14, 15]. Deviations in activity and feeding behaviour have only identified up to 58% of health issues successfully, while 55–71% of detected deviations did not co-occur with any known welfare issue [16, 17]. The reasons for the relatively poor detection of welfare issues remain unclear, which makes it difficult to pinpoint how it can be improved.

We propose three possible explanations. First, welfare-indicative behavioural deviations in individual pigs may not significantly exceed normal day-to-day variation, resulting in either too many or too few behavioural deviations being identified to accurately signal welfare issues. Supporting this, clear deviations were not detected in most individual pigs suffering from health issues [13]. That study assumed, however, that behavioural changes persisted throughout the welfare issue, while deviations may have occurred primarily upon or before issue onsets due to subsequent behavioural adaptation. Second, different aspects of behaviour (e.g. total daily duration, frequency or day-to-day repetition) may deviate in response to different welfare issues. Deviation detection algorithms, however, generally aim to detect any [e.g. 16, 18] or only specific welfare issues [e.g. 8, 11, 19, 20], hence possibly linking issues that do not induce deviations or detecting deviations indicative of unmonitored welfare issues. For example, while most individual pigs did not change their feeding behaviour during health issues, they did exhibit changes in feed intake, feeding duration and frequency during heat stress [13]. Finally, a pig’s baseline behaviour may influence its responses to welfare issues, as demonstrated in pigs with different physical characteristics or feeding strategies [13]. For example, a reduction in feeding frequency during lameness was only found for pigs with a high baseline feeding frequency (i.e. nibblers) [13]. Although individual baselines are commonly used in deviation detection [e.g. 16, 17, 18], if pigs with certain baselines do not change their behaviour when a welfare issue begins, this approach cannot improve detection.

This study aimed to provide more insight into how behavioural deviations relate to the onset of welfare issues in conventionally-housed growing-finishing pigs. Pig feeding behaviour was used as the behavioural welfare indicator as it is one of the few behaviours for which sensors are available for continuous recording in individual pigs [2, 3]; it has numerous established links to pig welfare [13, 14]; and it exhibits a variety of consistent individual baselines, referred to as feeding strategies [21–23]. We specifically studied (1) the relative frequency of detected deviations across behavioural variables, time, and individual animals; (2) the co-occurrence between deviations in different behavioural variables and any or a range of specific welfare issues; and (3) whether the co-occurrence between specific behavioural deviations and welfare issues improved or deteriorated in pigs with particular behavioural baselines (i.e. feeding strategies).

## Materials and methods

Data processing and analyses were performed in R, version 4.2.3 [24], unless stated otherwise. Figures were created using the *ggplot2* package [25] and tables using the *flextable* package [26]. Results are reported as mean ± standard error of the mean, unless specified otherwise.

### Animals and housing

This study followed four rounds of tail-docked growing-finishing pigs from a Topigs Norsvin (pig breeding company, The Netherlands) farm in Germany, as described in detail in [13] and Table 1. In short, each round included 110 barrows and/or gilts housed in 10 single-sex pens (11 pigs/pen, 440 pigs total) across 5 rooms. Observations spanned from arrival at the farm until the first or second group was sent to the slaughterhouse. Each pen had fully slatted floors, two drinking nipples providing *ad libitum* water, one IVOG^®^ electronic feeding station (Hokofarm group, The Netherlands) providing *ad libitum* access to pelleted commercial feed, and variable environmental enrichment (mix of chains, ropes, hosepipe and, in round 2, a crude fibre station providing chopped and pelleted straw). Rooms were naturally-lit with windows, and climate-controlled with mechanical ventilation and heating to maintain a target temperature that reduced from 27 to 22 °C across the growing-finishing phase.

**Table 1:** A description of the main characteristics that differed between the four rounds of pigs. Start and final weight (measured on-farm upon arrival and on the final weighing day) are given as the mean ± standard error, and the number of days in the barn reflects the day of arrival until the first (round 4) or second (rounds 1–3) group of pigs were transported to the slaughterhouse. The number (#) of pigs removed early either died (*n* = 1 in round 4) or were permanently moved to sickbay due to health issues. Medical treatments were only applied at farm level via the drinking water (unless pigs were in sickbay and out of the data collection). Table obtained from [11]

Characteristic	Round 1	Round 2	Round 3	Round 4
**No. of pigs**	110	109	110	110
**Pig breed**	Piétrain x (Landrace x Large White)	Landrace x Large White	Landrace x Large White	Tempo x (Landrace x Large White)
**Pig sex**	Half barrows, half gilts	Barrows	Barrows	Half barrows, half gilts
**Months**	Dec-Feb	Sep-Dec	May-Sep	Sep-Dec
**Days in barn**	92	92	100	83
**Start weight (kg)**	27.5 ± 0.3	24.7 ± 0.4	23.9 ± 0.4	25.9 ± 0.3
**Final weighing day**	75	76	78	76
**Final weight (kg)**	107.4 ± 0.8	106.1 ± 0.9	103.4 ± 0.7	108.8 ± 1.0
**No. of pigs removed early**	7	3	1	7
**Medical treatments**	-	-	-	2x Pulmodox (2 × 4d) & 1x Amoxicillin (6d)

### Data collection and processing

Data on pig feeding behaviour (Sect. 2.2.1) and welfare (Sect. 2.2.2) were collected to identify welfare issue onsets (Sect. 2.2.2.3) and determine pigs’ basal feeding strategies (Sect. 2.2.3).

#### Feeding data

Feeding data were collected throughout the growing-finishing phase using IVOG^®^ electronic feeding stations, as described in detail in [13]. In short, IVOG^®^ stations are single-spaced feeding stations that always have feed present in the trough, and record the timing, duration (s) and intake (kg) of each pig’s visit to the feeder. Electronic feeding station data were cleaned and aggregated to the meal level as described in [27] and summarised in the Supplementary Methods of [13]. In short, in pig rounds 1, 2, 3 and 4, respectively, in total 4.79%, 4.98%, 13.36% and 6.21% of visits were fully removed, and the intake and duration of an additional 6.81%, 6.18%, 17.59% and 12.61% of visits were removed. The calculated criteria for aggregation of visits into meals were 43, 61, 30 and 50 s in rounds 1, 2, 3 and 4, respectively (see Supplementary Methods of [13] for visualisations of the calculation).

Electronic feeding station data were used at the daily level, including the components feed intake, feeding duration, feeding frequency, feeding rate, proportion of intake obtained at night, and strength of the circadian rhythm in feed intake (onwards referred to as intake, duration, frequency, rate, night intake and circadian rhythm strength, respectively). A quantitative summary of the components is provided in Table 2 of [13]. Daily intake (kg) and duration (s) were calculated by summing meal intakes and durations, daily frequency by counting the number of meals, and rate by dividing the daily intakes by daily durations (g/s). Diurnal features were derived from hourly intake data (sum of meal intakes within the hour), including night intake and circadian rhythm strength. Night intake was calculated as the proportion of daily feed intake obtained between 21:00 and 03:59, which was the period before the morning intake peak started and after the afternoon intake peak ended, based on visualisations of the diurnal patterns per pig round and week. Circadian rhythm strength was measured using wavelet analysis as described in detail in [28]. In short, data were processed to meet analysis assumptions (i.e. de-trended, corrected for amplitude changes, and missing values were substituted with 0s), after which wavelet analysis was performed (Morlet base wavelet, continuous wavelet transform, periodicity range 8–48 h). The obtained periodicities between 23.5 and 24.5 h were extracted and subsequently aggregated to the daily level by calculating the median power of the day’s time points. To avoid edge effects, 2 d before and after days with missing data were removed. The result was a single dimensionless value for each pig day (i.e. day of an individual pig), where higher values indicated stronger circadian rhythms.

#### Welfare data

Data on pig welfare issues were obtained via on-farm health observations and climate sensors, from which health issues and heat stress data were extracted and reduced to a binary format (Sect. 2.2.2.1 and 2.2.2.2). Subsequently, from these binary data only the days surrounding issue onset were retained (Sect. 2.2.2.3).

##### Health issues

On-farm health observations were performed twice-weekly by a trained observer using the protocol presented in [29]. This protocol includes various pig-level and pen-level indicators, scored either as counts or on discrete scales ranging from binary to 0–5. In round 1, an older version of the protocol was used, which did not yet include conjunctivitis and lying bumps. For this study, only pig-level indicators were used as individual pig identification was required. An exception was coughing, which reached clinically-relevant levels during a pathogenic coughing outbreak in round 4 that was treated with Pulmodox (2 × 4 d) and Amoxicillin (1 × 6 d) in the drinking water. To include this outbreak, in each round a day was marked as a coughing day for all pigs in a pen if over fifteen coughs were counted during the 5 min-observation period [after 30].

Health issues were further selected based on relevance, reliability and additional value, leading to the exclusion of tear stains and lying bumps due to low relevance (both had very high occurrence and were unlikely to induce sudden responses), skin disease due to low reliability, and tail/ear/flank necrosis due to limited additional value beyond damage scores (i.e. scores showed similar trends). This process resulted in the inclusion of the following health issues: low body condition score, bursitis, conjunctivitis, coughing, blue ear disease, ear base damage, ear tip damage, flank damage, hernia, lameness, lesions on the front, middle or rear of the body, pumping, rectal prolapse, shivering and tail damage.

Discrete scores for these health issues were converted to a binary format, reflecting ‘issue-days’ (i.e. pig days upon which a welfare issue was scored), and ‘non-issue-days’. Classification thresholds were primarily based on literature. For example, a tail-biting outbreak is generally defined as ‘presence of a wound on the tail’ [31, 32], hence the threshold was set at the first score indicating a wound (i.e. score 2). When literature was lacking, thresholds were instead determined based on the distribution of the health data, to balance the number of days scored as with and without health issues. Supplementary Table S1 provides a quantitative summary of the health data, including original scores in total, per pig round, per month and per score level, and the thresholds used to convert them to binary format.

##### Heat stress

On days with high outdoor temperatures, maintaining the intended indoor temperature sometimes failed, causing heat stress. To identify heat stress days, environment-based and animal-based indicators were combined, as heat stress thresholds for pigs of specific body weights are unavailable in literature. Climate sensors were used to first identify days with a high temperature humidity index (THI), indicating a heat stress risk, after which panting observations were used to confirm that pigs were likely affected by the heat.

Ambient temperature and relative humidity were measured using a combined temperature and humidity sensor (iDOL114, DOL Sensors, Denmark) installed at approximately 1.8 m height in each room. These sensors are programmed to take a measurement every 15 min. As climate sensors were only installed from round 2 onwards, heat stress could only be identified during rounds 2, 3 and 4. Climate sensor data were visualised and cleaned to remove any temperature measurements below 16 °C and any humidity measurements below 40% or above 90% (i.e. unreasonable outliers based on visualisations). This cleaning process removed 15 temperature measurements from one sensor (0.05% of the data) in round 2, and 978 humidity measurements from one sensor (3.12% of the data) in round 4. The cleaned data were used to calculate the THI in each room for every 15 min, using Eq. 1 [33]. The 15 min-THI measurements were aggregated to the daily level for each room and then to the round level (i.e. aggregating all rooms), both by taking the median. A day was considered at risk of heat stress if the median THI exceeded 79 [based on 33, 34], indicating that the THI was above this threshold for half the day.

1 $$\begin{gathered}\:THI = \left( {1.8 \cdot \:AT + 32} \right) - \hfill \\\,\,\left( {\left( {0.55 - 0.0055 \cdot \:RH} \right) \times \:\left( {1.8 \cdot \:AT - 26} \right)} \right) \hfill \\ \end{gathered} $$

In which: THI = Temperature Humidity Index, AT = Ambient Temperature (°C) and RH = Relative Humidity (%).

Panting was initially scored at the individual level but appeared only reliable at group level, as panting varied with the pig’s body posture at the time of observation (i.e. more panting occurred in lying pigs than in those sitting, standing or in locomotion, results not shown). Therefore, the proportion of pigs panting at farm level was calculated for each observation day. Each day at risk of heat stress (i.e. with THI > 79) was marked as a heat stress day if ≥ 10% of pigs were panting, hence approximately ≥ 11 pigs in the barn. For non-observation days, the panting threshold was considered exceeded if it had been exceeded on the preceding or following observation day. A quantitative summary of the heat stress data is provided in Supplementary Table S1.

##### Identification of welfare issue onsets

Welfare data were processed to reflect only the onset of new bouts rather than all days with welfare issues. For all welfare issues except heat stress, this was done in three steps. In step one, welfare issue data were interpolated across non-observation days. Days were marked as issue-days if both neighbouring observation days were issue-days and as no-issue-days if at least one neighbouring observation day was a no-issue-day or missing. In step two, only days where the score switched from no-issue- to issue-days were retained, leaving only the onsets marked as issue-days. In step three, all days between the onset and the previous observation day (by definition a no-issue-day) were marked as issue-days, as the real onset may have occurred during this period. For heat stress, interpolation was unnecessary due to daily THI measurements, therefore only step two was applied to identify onsets (i.e. the first heat-stress-day following a no-heat-stress day). To illustrate this process: a pig was scored as sound on days 1 and 11 but as lame on days 4 and 8. In step one, days 5–7 are marked as lameness-days, and days 2–3 and 9–10 as non-lameness-days. In step two, only day 4, upon which the pig was first marked lame, is retained as a lameness-day. In step 3, this is extrapolated to days 2 and 3, as no scores were available for those days but the pig may have already been lame. Eventually, the pig’s lameness onset, as included for analysis, was denoted on days 2–4. Supplementary Table S2 details the onsets for each welfare issue, including the total number, the number of unique pigs and pens affected, the average, minimum, and maximum number of onsets per pig or pen, and the co-occurrence of onsets with other welfare issues.

Additionally, to assess if feeding behaviour deviations simply reflected any type of welfare issue, we created an ‘any issue’ variable based on the onset scores for individual welfare issues. Pig days were scored as any-issue-days if any kind of welfare issue had an onset on that day, and as no-issue-days if this was the case for none of them. In further analysis, this accumulation of all welfare issues was treated similarly to any individual welfare issue, and it is also quantified in Supplementary Table S2.

#### Determining feeding strategies

To understand how different feeding strategies impact feeding behaviour deviations upon welfare issue onsets, we studied deviations separately for groups with distinct feeding strategies. Four types of feeding strategies were included: nibbling/meal eating (based on frequency), fast/slow eating (based on rate), day/day-night eating (based on night intake) and consistent/inconsistent eating (based on circadian rhythm strength) [21–23, 28]. Although feeding strategies reflect continua of variation [21], for practical reasons pigs were classified into one of three categories (low, medium or high) for each feeding strategy. To perform this classification, first days with known or likely disturbances were removed from the feeding data: (1) all days before the first health observations; (2) all days after the first pigs were sent to slaughter; and (3) all days on which severe health issues were scored, with a 3 d-range around the observation day (the process is described in detail in the Supplementary Methods of [28]). Remaining data were scaled to eliminate time trends by subtracting the farm daily average and dividing by the farm daily standard deviation (function *scale()* [24]). For each pig and feeding component, a median value was calculated from the scaled data. Pigs with median values below the 33rd and above the 67th percentile of the pig round were assigned to the low and high categories, respectively, with the remaining pigs placed in the intermediate category. This approach ensured approximately equal numbers of pigs per feeding strategy group in each pig round, totalling 144 – 148 pigs per group.

### Data analysis

To explore how well sudden deviations in individual pig feeding behaviour co-occurred with the onset of welfare issues, pig behaviour was predicted day-by-day, generating alerts if observed behaviour deviated from these predictions (Sect. 2.3.1). Alerts were described (Sect. 2.3.2) and compared with pig welfare records, both for all pigs collectively (Sect. 2.3.3) and for subgroups of pigs with specific feeding strategies (Sect. 2.3.4).

#### Modelling pig behaviour day-by-day and generating alerts

**Fig. 1:**
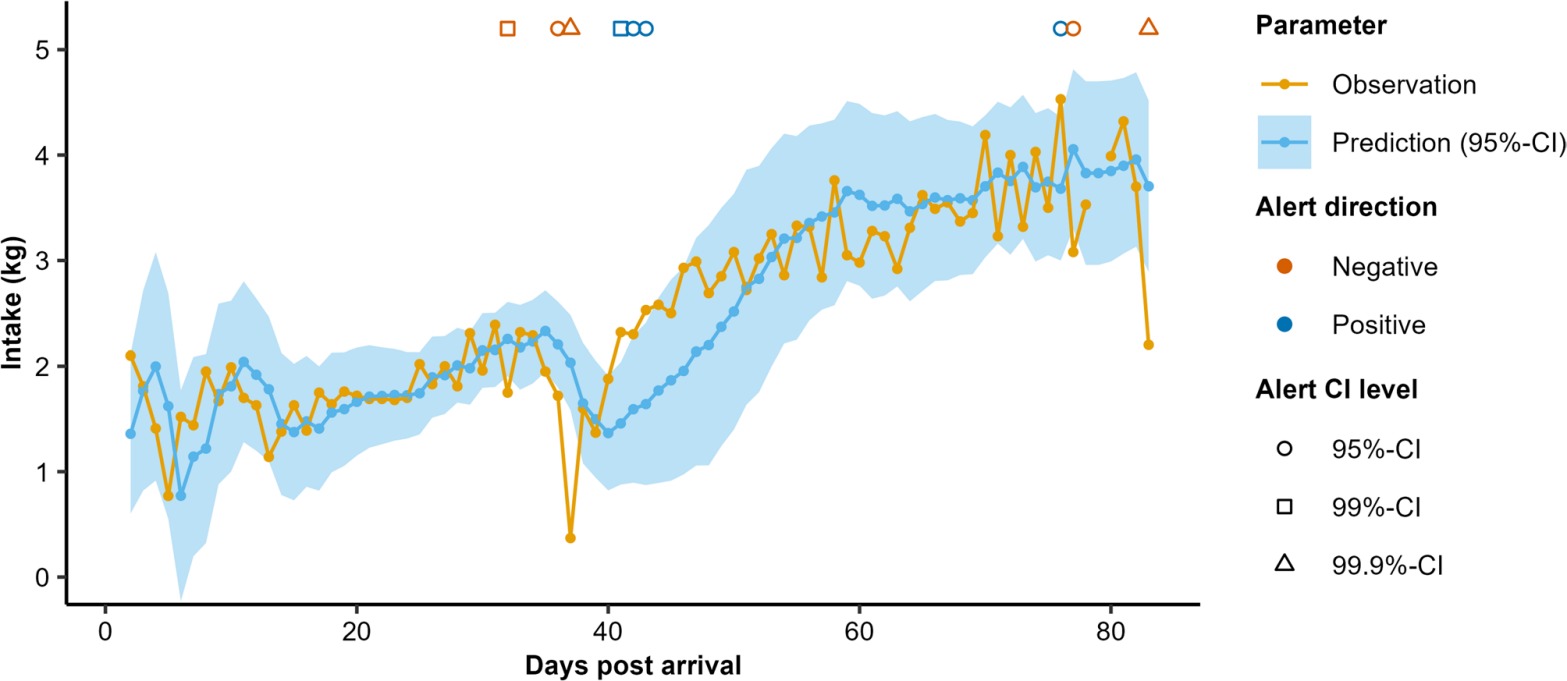
Example result of fitting the dynamic linear model with Kalman filter to the feed intake data of one pig, including predicted and observed values and different types of alerts generated (positive: too high feeding value, negative: too low feeding value, both at 95%-, 99%- or 99.9%-confidence interval (CI)). Only the 95%-CI is shown in shaded blue for figure readability

Dynamic linear models with a Kalman filter, fitted in MATLAB version 9.13.0 [35], were used to model each pig’s feeding behaviour components (i.e. intake, duration, frequency, rate, night intake and circadian rhythm strength) on a daily basis [after 36, 37]. Each day’s feeding component value was predicted using data from previous days, continuing until the last day of the pig’s time series, generally the end of the growing-finishing phase. Figure 1 provides a graphical illustration of the process.

##### Bases of the predictions

Predictions were made using a quadratic trend model that also incorporated the average behaviour of the pen, as formulated in Eq. 2–6. Descriptively, these equations state that predictions of each day were based on (1) the pig’s behaviour (i.e. feeding component value) on the previous day; (2) the trend in its behaviour; (3) the change in this trend; and (4) the average behaviour of the pigs in the pen, which could weigh more or less heavily across time.

2 $$\:{Y_t} = \mu {\:_t} + \gamma {\:_t} \cdot \:{\bar Y_t} + {v_t},{v_t} \sim \:N\left( {0,{V_t}} \right)$$

3 $$\:{\mu _t} = {\mu _{t - 1}} + {\alpha _{t - 1}} + {\beta _{t - 1}} + {w_{1t}}$$

4 $$\:{\alpha _t} = {\alpha _{t - 1}} + {\beta _{t - 1}} + {w_{2t}}$$

5 $$\:{\beta _t} = {\beta _{t - 1}} + {w_{3t}}$$

6 $$\:{\gamma _t} = {\gamma _{t - 1}} + {w_{4t}}$$

in which: Y_t_ = observed value of feeding component; $\:{\mu_t}$ = level of feeding component at time t; $\:{\gamma_t}$ = parameter for the influence of the pen average on the feeding component; $\:{\bar Y_t}$ = average of observed variable across all pigs in the pen; v_t_ = observational error, normally distributed with zero average and variance V_t_; $\:{\alpha _t}$ = linear trend at time t; $\:{\beta _t}$ = quadratic trend (i.e. change in trend) at time t; w_it_ = system error (i = 1,2,3,4).

##### Predicting on-line (day-by-day)

A dynamic linear model and Kalman filter were used to estimate the values of the variables in Eq. 2–6 on a day-by-day basis. This procedure has been previously described in [18, 36, 37] and is, for completeness, fully described in the Supplementary Methods of this manuscript. In short, the procedure estimated the ‘state’ of the system (i.e. the feeding behaviour component) at any given time t based on estimates that had been obtained and calculated at previous time steps. The procedure had two stages. In the prediction stage, the estimate of the coming state t was made. In the updating stage, the obtained estimate was updated with the real observation y_t_, and the estimation error e_t_ and its variance E_t_ were calculated. The estimates obtained during this process could subsequently be used to create alerts (Sect. 2.3.1.3) and to predict the system’s state (i.e. pig behaviour) for the next time step t + 1.

##### Generating alerts for deviations

The daily prediction of pig behaviour served to generate alerts for days when observed behaviour deviated from the prediction. A deviation was defined as an observed value lying outside the prediction’s 95%-, 99%- or 99.9%-confidence interval (CI). CIs were calculated using Eq. 7.

7 $$\:\left( {{\mu _t} + {\gamma _t} \cdot \:{{\bar Y}_t}} \right) \pm \:z \cdot \:\sqrt {{e_t}} $$

in which for 95%-CI, z = 1.96; for 99%-CI, z = 2.575, and for 99.9%-CI, z = 3.291. Alerts could not be produced on days with missing feeding station data, hence these days were marked as missing. Seven types of alerts were created: ‘any alert’ for when the observed value simply fell outside the 95%-CI, and ‘positive’ and ‘negative’ alerts for each CI level (i.e. 95%, 99% or 999.9%) when the observed value was higher or lower than the relevant CI, respectively.

##### Checking model fits and setting starting values

Model fits were assessed visually, in MATLAB, for each pig using plots of the predictions and generated alerts, such as in Fig. 1, and using plots of temporal patterns in the underlying variables $\:\alpha\:$, $\:\beta\:$ and $\:\gamma\:$. As this concerned ~ 5000 plots, checks were more for overall impressions than strict assessment of each individual model. Starting values for model variables, i.e. the values used on the first day of the time series, were iteratively refined based on these visualisations, to balance prediction validity, prediction CI width, and alert frequency. The final starting values used were: $\:\mu\:$ = 0.9 $\:\cdot\:$ first feeding component value; $\:\alpha\:$ = $\:\beta\:$ = 0; $\:\gamma\:$ = 0.1; and V_t_ = 0.1.

#### Description of alerts

Understanding when alerts were produced is essential for interpreting their co-occurrence with welfare issues, as it shows expected co-occurrences by chance and possible biases to address. Alerts were described in three different ways, always for each feeding component. First, the frequency of different alert types was assessed. For each component, all pig days were aggregated and the percentage of days with either no alert or any of the seven types of alerts was calculated. Second, alert distributions across months, rounds and pigs were determined. For each month and round, the percentage of pig days with positive and negative alerts was calculated. Additionally, the percentage of days with positive or negative alerts was determined for each pig, from which the average, standard error, minimum and maximum values across pigs were calculated. Third, it was assessed how often different feeding components generated alerts on the same day. For each feeding component and alert type, days with alerts were selected and the percentage of selected days with any type of alert for another feeding component was calculated.

#### Sensitivities for all pigs

Sensitivity is a proportional measure (0-100%) of how well onsets of welfare issues co-occurred with sudden deviations in feeding components, as marked by alerts. Sensitivities were calculated for each feeding component and welfare issue separately, in Microsoft Access. With our data collection and processing decisions, alerts could be produced daily while welfare issue onsets lasted 3–4 d (except for heat stress, which lasted 1 d). To align these, each onset’s alerts were aggregated into one value by retaining the most extreme alert. If alerts could not be created on more than a third of onset days due to missing feeding station data, the onset was excluded from further analysis (5.6% of onsets removed on average, range 0–32.3% across welfare issues, detailed in Supplementary Table S2). For each onset and alert type, onsets were classified as a True Positive if they co-occurred with an alert and a False Negative if they did not. Sensitivities were calculated using Eq. 8, but only for welfare issues for which an onset occurred in at least ten unique pigs, hence excluding blue ear disease, hernia, low body condition, pumping and shivering from analysis (see Supplementary Table S2).

8 $$Sensitivity = \:\frac{{True\,Positives}}{{True\,Positives + False\,Negatives}} \cdot \:100$$

Only sensitivities, and not measures such as specificity or positive/negative predictive value, were calculated for two reasons. First, with only twice-weekly on-farm observations and hence many days without (non-interpolated) welfare data, we considered the determination of False Positive (i.e. an alert occurred but no welfare issue was observed) or True Negative (i.e. no alert occurred and no welfare issue was observed) alerts unreliable. Second, our manuscript did not aim to test the validity of a welfare monitoring algorithm, for which these measures must certainly be considered beyond sensitivities, but rather aimed to use sensitivity as an indicator of possible co-occurrence between sudden changes in feeding behaviour and the onset of welfare issues. For this purpose, sensitivity measures are sufficient to identify interesting directions for future research, in which more measures should be included.

#### Sensitivities for pigs with specific feeding strategies

Sensitivities were also calculated for each subgroup of pigs with a specific feeding strategy (i.e. low, intermediate or high in nibbling/meal eating, fast/slow eating, day/day-night eating and consistent/inconsistent eating, Sect. 2.2.3) using the same methods as for all pigs collectively (Sect. 2.3.3). For practical reasons, only a selection of welfare issues was analysed, representing various types of issues with distinct behavioural deviations noted in this study or in [13]: flank damage, tail damage, lameness and heat stress. Sensitivity calculations were only performed for subgroups with at least ten unique pigs with an onset, but this did not result in any exclusions of subgroups or welfare issues (Supplementary Table S3).

## Results

### Description of alerts

Missing days concerned 8.6% of pig days for feeding components duration and frequency, 13.4% of pig days for intake, rate and night intake, and 29.2% of pig days for circadian rhythm strength. Results are hereafter reported after removal of these missing days, i.e. the total number of pig days (100%) is after deduction of missing days.

**Fig. 2:**
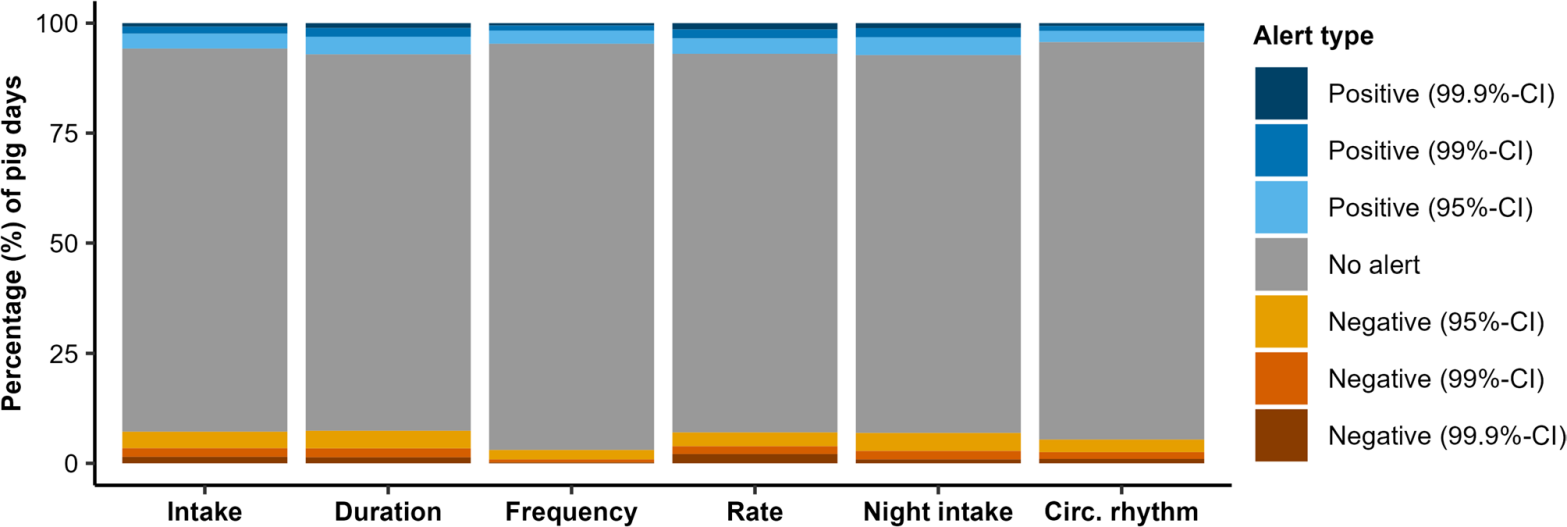
Per feeding component, the percentage of pig days upon which an alert was created by the dynamic linear model with Kalman filter. Alerts could indicate an increase (‘Positive’) or decrease (‘Negative’) in the feeding component, and were created using 95%-, 99%-, and 99.9%-confidence intervals (CI)

Figure 2 shows the percentage of pig days upon which different types of alerts were obtained for each feeding component. Overall, distributions of alert types were similar across the feeding components, though the total number of alerts differed. More alerts were created for intake, duration, rate and night intake (alerts on average on 14.5% of days) than for frequency and circadian rhythm strength (alerts on average on 7.7% of days). By definition, there were relatively more alerts at 95%-CI, followed by 99%-CI and finally 99.9%-CI, but this was also the case for how many *extra* alerts were generated at wider CIs. For practical reasons, hereafter results are only reported for the 95%-CI (i.e. including alerts that were also outside the 99%-CI or 99.9%-CI). During explorations with the other thresholds, similar patterns as for the 95%-CI were observed (results not shown).

Table 2 quantifies the alerts. For most feeding components, there were fewer alerts in the first than in later months for both negative and positive alerts. This difference was especially noticeable for rate and circadian rhythm strength. Exceptions to this increasing pattern occurred for duration, which had more positive and negative alerts in month 1 than months 2 and 3, and for frequency, which had more positive and negative alerts in months 1 and 2 than in month 3. There was little difference in alert frequencies between different pig rounds for all feeding components, though some variation was present. For individual pigs, alert frequency differences were quite prominent, ranging from 1.1 to 2.2% for the lowest to 11.6 – 22.2% for the highest alert frequency across feeding components.

**Table 2:** Quantifications of the alerts created by the dynamic linear model with Kalman filter

Feeding component	Total alerts		Month			Round				Pig		
	*#*	*%*	*1*	*2*	*3*	*1*	*2*	*3*	*4*	*Mean ± SEM*	*Min*	*Max*
***Negative (95%-CI)***												
Intake	2,428	7.2	4.5	8.4	8.6	6.9	6.8	7.5	7.6	7.3 ± 0.1	1.3	16.1
Duration	2,612	7.4	8.9	6.5	7.0	7.1	7.3	7.0	8.3	7.5 ± 0.1	2.2	22.2
Frequency	1,069	3.0	3.5	3.9	1.7	3.4	3.6	2.3	2.7	3.4 ± 0.1	1.1	22.2
Rate	2,372	7.0	1.1	8.9	10.9	7.1	6.9	7.1	6.9	7.0 ± 0.1	1.6	14.5
Night intake	2,332	6.9	5.4	8.3	6.9	6.9	7.5	5.8	7.4	6.9 ± 0.1	1.4	14.1
Circ. rhythm	1,605	5.4	2.1	6.5	8.0	6.0	5.6	4.1	5.7	5.5 ± 0.1	1.2	16.7
***Positive (95%-CI)***												
Intake	1,951	5.8	3.4	7.5	6.3	5.8	5.5	5.5	6.4	5.8 ± 0.1	1.1	12.7
Duration	2,506	7.1	8.2	6.8	6.4	7.2	6.7	6.5	8.1	7.2 ± 0.1	1.1	22.2
Frequency	1,654	4.7	4.2	4.7	5.2	4.0	4.4	5.5	4.8	4.8 ± 0.1	1.1	11.6
Rate	2,357	7.0	1.0	9.3	10.4	7.0	7.1	7.0	6.7	6.9 ± 0.1	1.6	14.1
Night intake	2,459	7.3	5.2	8.7	7.8	7.0	7.2	7.3	7.7	7.3 ± 0.1	1.2	14.3
Circ. rhythm	1,281	4.3	1.7	4.8	6.8	4.6	4.1	3.4	5.2	4.6 ± 0.1	1.1	13.9
All values reflect the percentage of pig days with an alert, except for the total alert count which is given in both absolute number (#) and percentage (%) of pig days. Values are provided for positive and negative alerts (both at a 95%-confidence interval (CI)) and each feeding component (‘Circ. rhythm’ = Circadian rhythm strength)												

All values reflect the percentage of pig days with an alert, except for the total alert count which is given in both absolute number (#) and percentage (%) of pig days. Values are provided for positive and negative alerts (both at a 95%-confidence interval (CI)) and each feeding component (‘Circ. rhythm’ = Circadian rhythm strength)

**Fig. 3:**
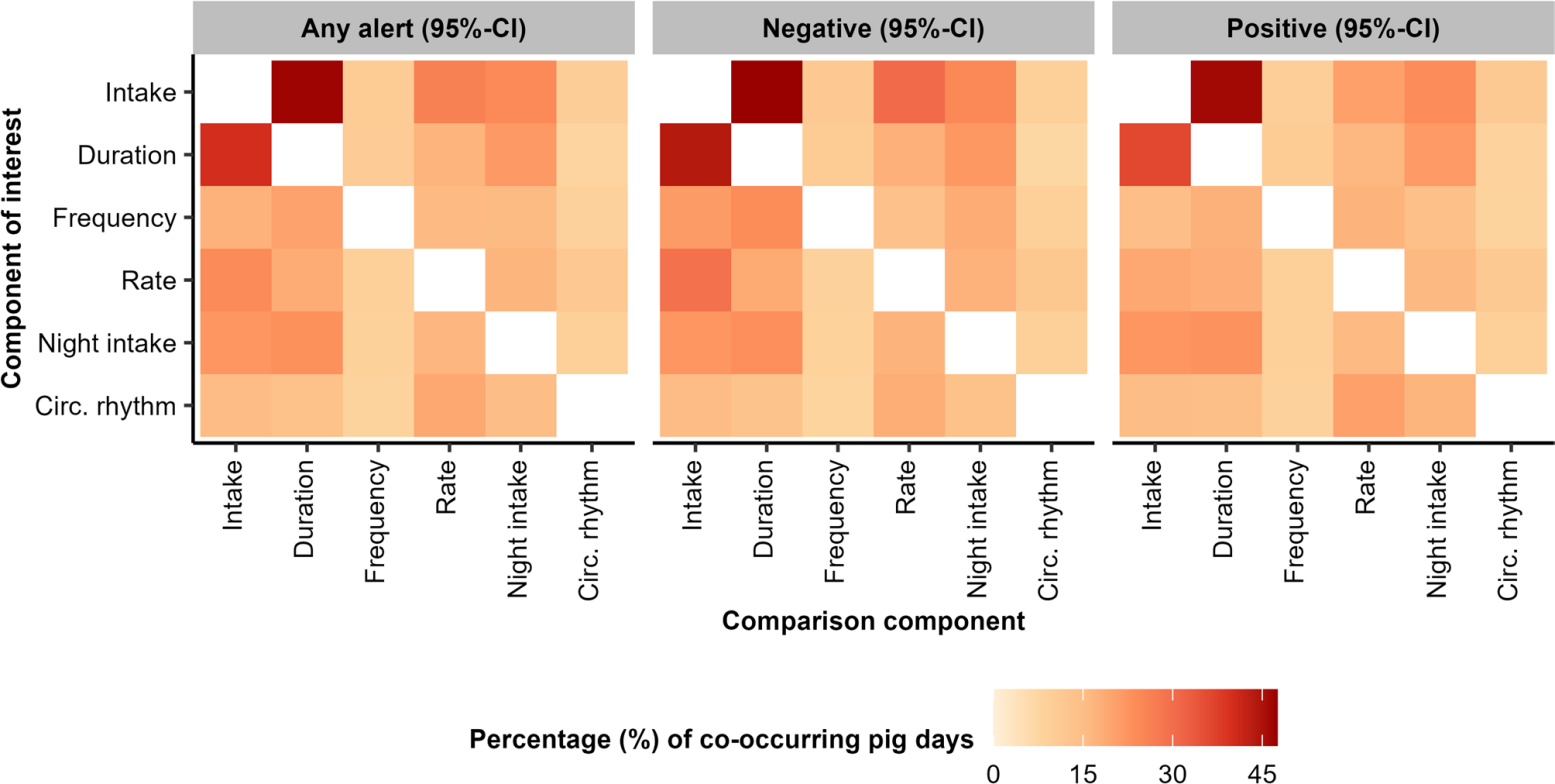
A heat map of the daily overlap in alerts (at a 95%-confidence interval (CI)) between different feeding components. For each feeding component of interest, the fill colour represents the percentage of alert days (either any alert, a positive one or a negative one) upon which the comparison component also had any type of alert. For example, out of all days with an alert for intake, 47% also had an alert for duration, while out of all days with an alert for duration, 40% also had an alert for intake

Figure 3 shows the daily overlap in alerts between different feeding components. When any type of alert was considered, the percentage of days upon which another feeding component also had an alert was on average 18 ± 2%, ranging from 8 to 47% between feeding components. The highest degree of overlap occurred between duration and intake, and the lowest degrees of overlap were with frequency and circadian rhythm strength. When looking at only negative or only positive alerts, similar distributions (Fig. 3), averages (positive alerts: 17 ± 2%; negative alerts: 18 ± 2%) and ranges (positive alerts: 8–46%; negative alerts: 7–48%) were seen.

### Sensitivities for all pigs

**Fig. 4:**
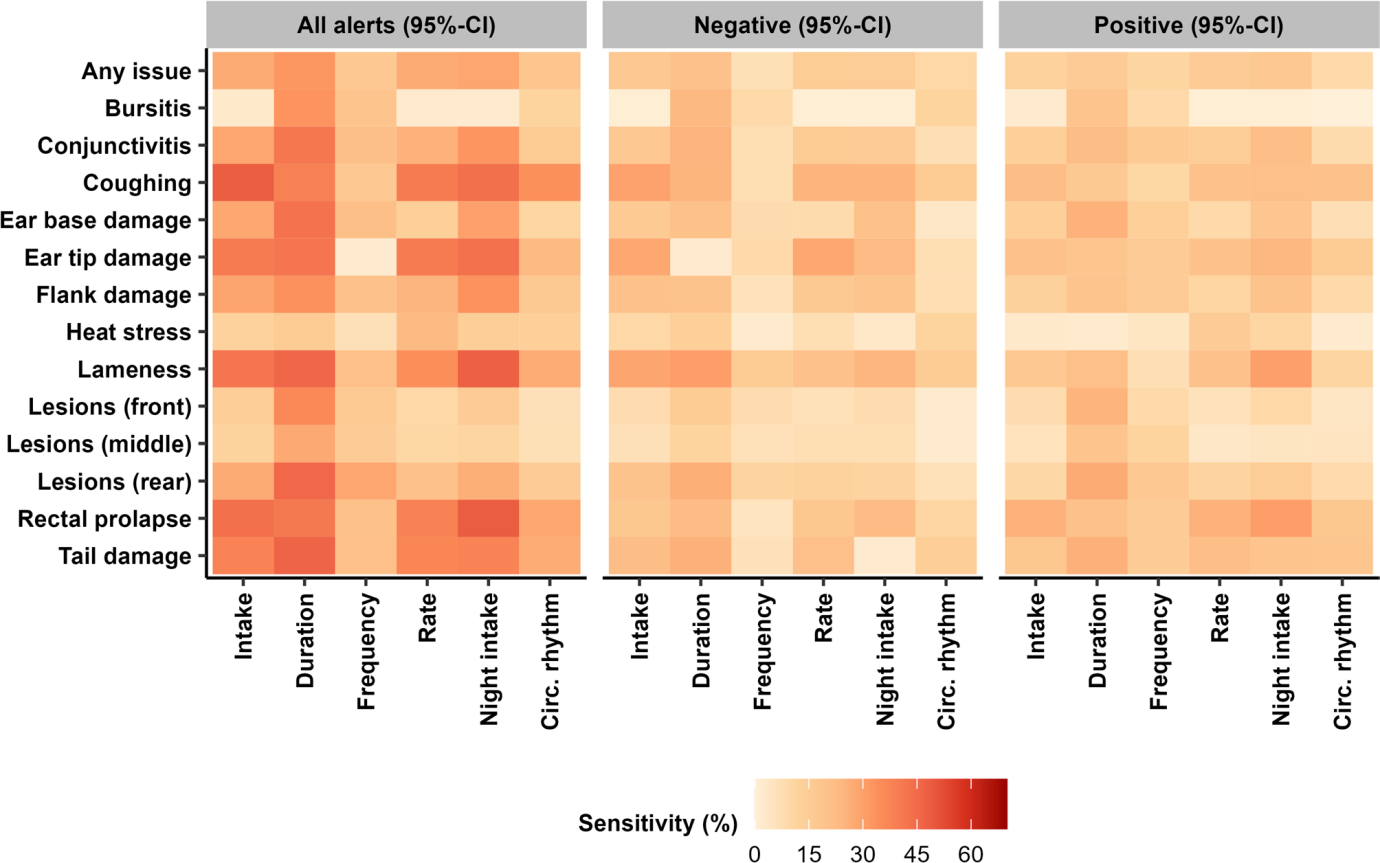
A heat map of how well the alerts created upon sudden deviations in the feeding components correspond with the onsets of different welfare issues, i.e. their sensitivities. Alerts reflect either all alerts or only those indicating negative or positive deviations, always at a confidence interval (CI) of 95%

The sensitivities that reflect the agreement between feeding component alerts and welfare issues are shown in Fig. 4. In general, sensitivities were low, ranging from 0.0 to 48.7%, with relatively higher sensitivities for all alerts (range 2.0 – 48.7%) than for only negative (range 1.0 – 33.3%) or positive (range 0.0 – 30.8%) alerts. In general, relatively higher sensitivities were obtained for feeding components duration, night intake, intake and rate than for other components, especially when all alerts were considered. In addition, relatively higher sensitivities were also obtained for welfare issues coughing, ear tip damage, lameness, rectal prolapse, and tail damage than for other welfare issues. The highest sensitivities obtained (45–50%) matched sudden changes in night intake to rectal prolapse; intake to coughing; night intake or duration to lameness, and duration to lesions on the rear part of the body.

### Sensitivities for pigs with specific feeding strategies

**Fig. 5:**
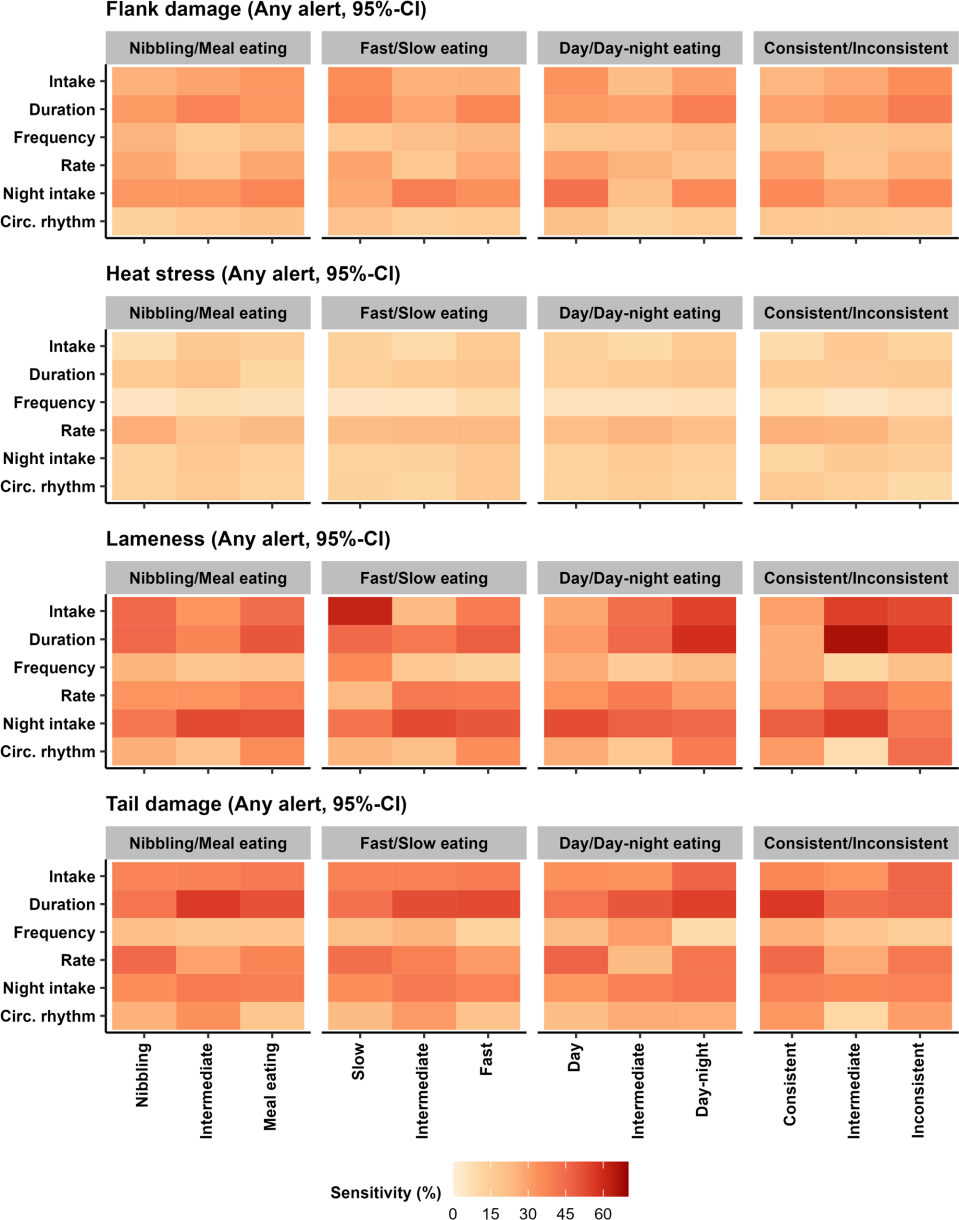
For subgroups of pigs with different feeding strategies, a heat map of how well alerts indicating suddenly deviating feeding patterns (at a 95%-confidence interval (CI)) corresponded with each of four example welfare issues (i.e. alert sensitivity)

Figure 5 shows how well alerts co-occurred with onsets of flank damage, tail damage, lameness and heat stress for subgroups of pigs with specific feeding strategies. The sensitivities generally remained low but could become relatively higher for specific subgroups (range: 4.6–66.7%), resulting from sensitivity changes that ranged between − 19.2 – +20.2% (for the exact changes, see Supplementary Figure S1). In general, sudden changes in feeding behaviour still co-occurred poorly with onsets of flank damage (sensitivity range changed with − 12.5 – +9.6% to 12.9 – 43.2%) and heat stress (with − 5.2 – +4.7% to 4.6 – 26.9%), regardless of which feeding strategy subgroup was considered. Some subgroup sensitivities did increase for lameness (with − 19.2 – +20.2% to 8.3 – 66.7%) and, to a smaller extent, for tail damage (with − 16.7 – +10.3% to 9.2 – 56.9%).

The largest improvements in sensitivities were seen for lameness, which more frequently co-occurred with sudden changes in (1) intake in slow eaters (with + 19.9 to 61.9%), day-night eaters (with + 12.5 to 54.5%), and more inconsistent eaters (intermediates: with + 13.5% to 55.6%, inconsistent: with + 10.9 to 52.9%); (2) duration in day-night eaters (with + 12.6 to 59.1%) and more inconsistent eaters (intermediates: with + 20.2 to 66.7%, inconsistent: with + 11.4 to 57.9%); (3) frequency in slow eaters, though overall sensitivity remained relatively low (with + 15.2 to 36.4%); 4) night intake in more meal-eating pigs (intermediates: with + 5.5% to 53.3%, meal eaters: with 3.9% to 51.7%), day eaters (with + 4.6% to 52.4%), and more consistent eaters (intermediates: with + 7.7 to 55.6%, consistent eaters: with + 0.7 to 48.5%); and (5) circadian rhythm strength in day-night eaters (with + 12.7 to 40.0%) and inconsistent eaters (with + 17.2 to 44.4%).

For tail damage, sudden changes in duration more often co-occurred with onsets of welfare issues in more meal-eating pigs (sensitivity of intermediates: changed with + 9.3 to 56.2%, meal eaters: with + 4.8 to 51.7%), faster eaters (intermediates: with + 5.6 to 52.5%, fast eaters: with + 6.0 to 52.9%), day-night eaters (with + 8.4 to 55.4%), and consistent eaters (with + 10.0 to 56.9%). For intake, sensitivities also increased in day-night eaters (with + 8.9 to 46.9%) and inconsistent eaters (with + 8.5 to 46.6%), and for rate sensitivities increased in nibblers (with + 9.1 to 46.0%), day eaters (with + 10.3 to 47.3%) and consistent eaters (with + 8.6 to 45.6%).

## Discussion

This study aimed to provide more insight into how detected deviations in growing-finishing pig feeding behaviour relate to the onset of welfare issues, specifically health issues and heat stress. It (1) described the frequency of detected deviations (i.e. alerts) at different reliability thresholds (i.e. 95%-, 99%- and 99.9%-CI) in increasing (i.e. positive) and decreasing (i.e. negative) directions. Variation in alert frequency was also quantified across feeding components, months of the growing-finishing phase and individual pigs. Subsequently, (2) the co-occurrence between alerts and the onset of welfare issues was quantified by calculating the sensitivity. (3) This co-occurrence was also calculated for subgroups of pigs with specific feeding strategies.

### Alert frequency distributions (aim 1)

Alerts were generated at relatively high frequencies: between feeding components, on average once every seven to thirteen days (i.e. 7.7 – 14.5% of days had an alert). Frequencies were relatively higher for intake, duration, rate and night intake than for frequency and circadian rhythm strength (Fig. 2; Table 2). These differences were consistent across different CI levels and between positive and negative alerts. In addition, alerts in one component generally were not duplicated in another component on the same day (18 ± 2% of alerts were duplicated). This was even for components intake and duration, which are known to be highly correlated (i.e. correlation coefficients between 0.3 and 0.7 depending on age [38–42]). These findings suggest that sudden behavioural deviations occur more often in some feeding components than others, possibly in response to different events. A simple interpretation could be that pigs are more persistent in their frequency and circadian rhythm strength than for other feeding components, which is supported by both constituting relatively strong feeding strategies [21, 22, 28]. However, the frequency of alerts is not only determined by the size of the behavioural deviation, but also by the basal day-to-day consistency in the behaviour. A generally low day-to-day consistency results in a wider CI, from which only relatively large deviations can be detected. A generally high day-to-day consistency, in contrast, narrows the CI and enables detection of smaller deviations. Therefore, it could also be that the balance between day-to-day consistency and deviation size differed between components rather than the frequency of behavioural deviations themselves. Based on visualisations in individual time series (not shown), we expect that for frequency the lower alert frequency is due to a high day-to-day consistency combined with smaller deviations, while circadian rhythm strength has a low day-to-day consistency that masks possible deviations.

The relatively small differences in alert frequencies between rounds suggests that the frequency of deviations is little impacted by season and breed differences. This supports the idea that alert frequencies are more dependent on model settings than on-farm differences. Indeed, for most feeding components, alerts occurred more frequently in months 2 and 3 than in month 1; an expected pattern as dynamic linear models require several data points, i.e. days, to adapt the arbitrary starting values to individual pig data, resulting in larger CIs at the beginning of the time series. Statistical scaling effects [e.g. 43] could also make deviation detection more sensitive in some months than others. This is, however, unlikely to explain the full variation as the different feeding components have different, sometimes opposing, temporal trends (e.g. linear increase in feed intake compared to initial increase but predominantly a decrease in feeding duration). A biological explanation could be that pigs’ feeding behaviour is more unstable and variable from day-to-day when pigs are younger [21, 28] or still habituating to the farm, hence statistically masking relevant deviations. In addition, it could be that more deviations in later months reflect a higher frequency of welfare issues at older ages, however this is only scarcely supported by the slight increase in welfare issues in month 3 compared to months 2 and 1 (Supplementary Table S1). In contradiction, different patterns were seen for duration, which had more alerts in month 1 than in months 2 and 3, and for frequency, which had more alerts in months 1 and 2 than in month 3. Possibly, the convergence of the CI width was subordinate to age developments in the pigs’ behaviour. As pigs grow larger, their feeding frequency and duration reduce [14, 38, 39, 44, 45] and their individual feeding strategies develop [21, 22, 28]. This could have limited pigs in deviating their behaviour at later ages, when reducing duration and frequency further was too costly for maintaining sufficient feed intake, or when pigs had found an effective behavioural strategy that they did not want to deviate from.

Interestingly, there was a large range in alert frequencies between individual pigs, ranging between 1.1 and 22.2% of days with a positive or negative alert between feeding components. To our knowledge, individual alert ranges have not been previously reported in any animal species, though many studies in pigs have reported that variation in feeding behaviour is moderately heritable [46–50]. If individual differences in alert frequency are consistent across feeding components, this would indicate that some pigs show more or clearer behavioural deviations than others. This may, for example, depend on their feeding strategies, how frequently they encountered health issues, their dominance ranking or their genetics. Alternatively, it could reflect a statistical artefact, where this alert range is simply the deviation around the mean, especially if individual alert frequency is inconsistent across feeding components. A quick exploration (see Supplementary Figures S2-5) suggests that alert frequency is not consistent across feeding components and does not relate to feeding strategies or frequency of health issue onsets. Whether the variation is biologically relevant or represents a statistical artefact remains unclear and requires further study.

### Co-occurrence between alerts and welfare issues (aims 2 & 3)

As mentioned previously, alerts were generated at relatively high frequencies, with 7.7 – 14.5% of days marked as deviating (Fig. 2; Table 2). This high frequency corresponds well with the frequency of welfare issue onsets, as the more than 1500 onsets observed equated to approximately 14% of pig days affected (Supplementary Table S2). Nevertheless, this correspondence also implies that the odds of obtaining a match between an alert and a welfare issue onset, simply by chance, are quite large. For health issues (not heat stress), the odds increased further after aggregation of alerts obtained at specific days to 3–4 d periods. Overall, these numbers imply that a sensitivity of approximately 7.7 – 14.5% for heat stress (i.e. the same as the alert frequency) and 21 – 47% for health issues is to be expected between feeding components (i.e. 1 - (1–8.7/100)^3 on the low end for onset aggregation to 3 d, and 1 - (1–13.9/100)^4 on the high end for onset aggregation to 4 d).

#### Health issues

For health issues, sensitivities ranged from 2.0 to 48.7%, hence correspondence between detected deviations and welfare issue onsets was poor and, notably, not better than expected by chance. These sensitivities are poorer than what was previously obtained for detecting disease from feeding behaviour or activity in fattening pigs [16, 17, 20, 51, 52] and dairy cattle [53–57]. Nevertheless, they correspond with previous results on these same experimental data that showed that pigs were highly inconsistent in whether (and how) they changed their feeding behaviour during welfare issues [13]. Our results, both in this and our previous study [13], suggest that (sudden) deviations in feeding behaviour at pig level do not validly correspond with (onsets of) health issues.

This low correspondence could partly be a consequence of our definition of a health issue onset as the 3–4 d before first observation, or the relatively mild criteria for health issues used. A simulation study previously found that, although disturbances (i.e. welfare issues) could be reliably identified especially at farm and pen level, the day upon which disturbance onset was detected was generally later than the true onset, especially with longer-lasting disturbances [58]. It could hence be that behavioural deviations can primarily be picked up (shortly) after issue onset, and this could also differ between feeding components. Similarly, previous studies on feeding behaviour deviations typically compared to more extreme welfare issues or long-lasting welfare issues than in our study, such as mortality or medical treatments [46, 48, 58] and drops in production efficiency [47, 49, 50]. In addition, disturbances affecting the whole pen or farm were, in simulation, more reliably identified than those affecting only individual pigs [58]. It hence may be that the primarily pig-focused welfare issues in this study were too mild to induce sudden feeding behaviour deviations. However, more severe tail and flank damage were not observed to induce consistent long-lasting deviations in these data, either [13]. It should be noted that using a longer time frame or stricter criteria would only benefit sensitivities if the number of alarms is also drastically reduced. Such a reduction could potentially be achieved by changing the settings and starting values of the dynamic linear model, increasing the reliability threshold of alerts (i.e. taking a wider CI) or by requiring alerts in at least two feeding components before marking a day as behaviourally deviating. Unlike most other studies, we did not focus on optimising model performance, because our main aim was to look at relative differences in sensitivity across feeding components and welfare issues and not to develop a valid welfare issue detection algorithm. Improving the sensitivities with these steps would also reduce the detail in which welfare can be monitored and allow identification of only the most extreme welfare issues.

Sensitivities further deteriorated when only positive or negative alerts were considered (Positive: 0.0 – 30.8%; Negative: 1.0 – 33.3%), indicating that the direction of a behavioural deviation was irrelevant to its link with welfare issues. From a theoretical perspective, this is surprising, as a certain directionality in responses is to be expected for some combinations of feeding components and welfare issues. For example, most diseases should reduce feed intake rather than increase it [14, 32, 59], while feeding frequency could be reduced by lameness [60] and increased by being tail-bitten [61]. The lower sensitivities of positive and negative alerts for all combinations of welfare issues and feeding components were, however, not surprising considering their fifty/fifty contribution to the alert frequency. Previous studies focused primarily on only negative deviations [46, 49], which would not have worked for all feeding components in this study, or identified responses to disturbances without differentiating between positive or negative deviations [47–49, 58]. It could be that deviations in both positive and negative directions reflect both the initial behavioural deviation and the subsequent return to baseline. However, considering the alert frequencies and poor sensitivities, it is more likely that the alerts represent a statistical probability of deviation detection regardless of the direction. This corresponds with our previous findings on these same data, in which pigs equally changed their feeding behaviour in positive and negative directions during welfare issues [13].

We had hypothesised that behavioural deviation detection models could only indicate certain aspects of welfare, as only specific welfare indicators would associate with the behavioural deviations. For example, previous studies showed that lameness but not diarrhoea associated with reduced feeding duration and that skin lesions associated with reduced feeding frequency but not duration [14, 62]. In this study, we indeed found a range in sensitivities depending on the considered feeding components and welfare issues (Fig. 4). The highest sensitivities were obtained not for the accumulation of all welfare issues (i.e. ‘any issue’) but rather for combinations of coughing, ear tip damage, lameness, rectal prolapse and tail damage with intake, duration or night intake. However, these sensitivities were never higher than could be expected by chance, nor were they much better than when any type of welfare issue was considered. Therefore, our results provide some indication of health issues that may be better detected with specific feeding components, but cannot confirm their effectiveness definitively.

When feeding strategies were also considered, associations between deviations in certain feeding components and specific welfare issues became more evident and sometimes exceeded what was expected by chance (range: 4.6 – 66.7%). Specifically, higher sensitivities were obtained for detecting lameness based on intake and/or duration for slow eaters, day-night eaters and (intermediately) inconsistent eaters, and based on night intake for meal eaters, day eaters and (intermediately) consistent eaters. In addition, there were some smaller improvements for detecting tail damage from duration in more meal-eating pigs, faster eaters and consistent eaters. Note that these associations do not imply that pigs ate faster/slower, in larger/smaller meals, more/less at night or more consistently/inconsistently *during* these welfare issues. Rather, pigs with those specific feeding strategies in the *absence* of detected welfare issues were more likely to deviate their feeding behaviour *during* the onset of specific welfare issues. The identified groups largely correspond with our previous findings [13]. In that previous study, however, no groups were identified changing night intake during lameness, and additional changes in frequency were seen for pigs with similar feeding strategies, which here were limited to improved sensitivities that remained low (e.g. frequency in lame slow eaters). Possibly, this is due to the different methodologies applied, where the present study focused on sudden changes while our earlier study [13] focused on persistent ones (also see next section on heat stress). Overall, these findings confirm the relevance of pig feeding strategies for behavioural changes, across statistical methods, and hence further stress the importance of incorporating variation in pigs’ basal behaviour when trying to identify welfare-relevant deviations. Nevertheless, this should be further confirmed on an independent dataset, and the causal direction of these relationships should be determined in an experimental rather than observational study.

#### Heat stress

For heat stress, sensitivities ranged from 6.4 to 23.2% and were hence very poor, though they reached slightly better levels than could be expected by chance. As with health issues, discriminating between positive and negative alerts did not improve but rather deteriorated performance (Positive: 1.9 – 15.8%; Negative: 2.1 – 13.9%), and there were no pigs with specific feeding strategies for which sensitivities improved markedly. The particularly poor sensitivities, compared to those obtained for health issues, were due to a lack of 3 – 4 d-aggregation of heat stress onsets, meaning alerts were only classified as ‘true’ if they occurred on the single day of heat stress onset. If such 3 d-aggregation was performed, sensitivities ranged from 14.6 to 46.7%, similarly to health issues (more specific results not shown).

The poor sensitivities for heat stress across feeding components were in stark contrast with our expectations. In literature, the changes in pig feeding behaviour during heat stress are well-documented [14, 63–65]. Moreover, in our previous research on the same dataset, heat stress was the only welfare issue for which very consistent individual reductions in feeding activity were identified [13]. The discrepancy in findings may be due to methodological variations between the studies. In previous studies, effects of heat stress on (individual) feeding behaviour were recorded as longer-term behavioural changes that sustained throughout the heat stress period [13, 63–65]. In this study, however, we only measured *sudden* behavioural changes that co-occurred with heat stress *onset*. It hence seems that there are different types of behavioural deviations, with some being more sudden or persistent than others. In the case of heat stress, individual reductions in feeding activity seem to be gradual, not sudden, and persistent across the period of heat stress. This distinction between deviation types is highly relevant for the future development of welfare monitoring algorithms, as different methods may be required to detect different types of deviations [e.g. time-point detection such as in this study versus gradual-change detection such as developed in [66, 67, 68]].

### Implications for welfare monitoring

This study ultimately aimed to support the development of an algorithm that detects welfare issues in individual pigs, by identifying the most promising feeding components and welfare issues for further exploration. Such an algorithm could, for example, be used for early detection and intervention, for retrospective assessment of welfare issues to improve management or genetic selection, or to gain a better understanding of how different welfare issues affect individual pigs. Our findings, however, indicate that behavioural deviations are not a reliable indicator of emerging welfare issues in most pigs. Therefore, detection of such behavioural changes may be of little value for a welfare monitoring algorithm. Instead, it may be more valuable to further study longer-term, more gradual changes from expected behaviour, such as were detected for heat stress in [13], or general variation in feeding behaviour that is not day-specific [e.g. 48, 50]. These would be especially relevant in retrospective monitoring rather than for early-warning systems, aiming to, for example, improve pig housing and management or breed for resilience [e.g. benefits for next generation, [48]]. In addition, it may be worthwhile to study other, non-feeding-related behaviours that are more easily compromised during mild welfare issues, such as play or positive social interactions. Finally, combining volatile behavioural indicators with physiological indicators that are more stable from day-to-day, such as pig body weight, may be an interesting path.

The better correspondence between behavioural deviations and health issues in pigs with specific feeding strategies suggests that there are certain types of pigs for which deviations in feeding behaviour can indicate welfare issues. Studying these individual basal behaviours may reveal additional promising avenues to achieve welfare monitoring. Considering the impact of the housing environment on pigs’ feeding behaviour [14, 69–71], an interesting pathway could be to try and manipulate pigs’ basal feeding behaviour by changing their environment. Pigs could be stimulated to develop more consistent day-to-day patterns by, for example, reducing competition for the feeder and giving them more space and other resources to perform a wider range of species-specific behaviours. From more consistent patterns, welfare-relevant deviations may become more evident and easier to detect. Moreover, such enriched housing conditions could also provide a basis from which positive welfare or affective states could be incorporated into welfare monitoring algorithms, hence going beyond the current focus on welfare issues to gain a better representation of pig welfare [4]. For example, behaviours that are more likely to be expressed in enriched environments, such as play or positive social interactions, could be studied in detail, studying the individual variation in their basal patterns and their links to welfare indicators.

### Study limitations

The feeding and welfare data were subjected to many cleaning, aggregation and other processing choices before analysis, the most evident of which were threshold determinations and interpolation steps. These choices were necessary to enable analysis, but may have introduced biases in the data (e.g. some pigs had more feeding data removed than others, welfare issue thresholds could have been placed at more or less severe levels). All choices for data processing were made a priori based primarily on literature and if necessary on data distributions, and were similar to our previous study on these data [13] to facilitate comparison. In that previous study, different choices in data processing were never seen to change the main results (unreported results). To maintain comparability and in light of the explorative nature of this study, dynamic linear model performance was visually optimised but its correspondence to the welfare issue onsets was not. As such, the absolute values of the sensitivities should not be interpreted directly – rather, the focus of this manuscript lies on the relative differences in sensitivities between different feeding components, welfare indicators and feeding strategies. Finally, it should be noted that the results were obtained in a specific production system in one growing-finishing farm and, as feeding behaviour is strongly influenced by pig housing conditions [reviewed by 14], the results may change in other housing circumstances.

## Conclusion

We found that the frequency of behavioural deviations, indicated by model alerts, was relatively higher in intake, duration, rate and night intake than in frequency and circadian rhythm strength. Across feeding components, deviations occurred on different days, and their frequency was little influenced by rounds, generally increased across time, and varied largely between individual pigs. Together, these findings suggest that different feeding components indicate different, complementary behavioural deviations, and that their interpretations should be corrected for biases with age and pig differences. We also found that the co-occurrence of behavioural deviations with the onset of welfare issues or heat stress was not better than could be expected by chance, and that deviations’ directionality seemed unrelated to welfare issue onsets. The best co-occurrences were found for deviations in intake, duration or night intake with coughing, ear tip damage, lameness, rectal prolapse and tail damage, but these were still poor. Co-occurrences exceeding chance expectations were seen for intake, duration and night intake in tail-bitten or lame pigs with specific feeding strategies, suggesting pigs change their responses to welfare issues depending on their normal feeding behaviour. The particularly poor co-occurrence of heat stress onsets with feeding behaviour deviations suggests that there are different types of deviations, where those during heat stress are not sudden but rather gradual and persistent. Overall, our results suggest that detection of welfare issues based on individual pig feeding behaviour is difficult, but may be mildly effective for specific welfare issues and specific types of pigs. Considering the impact of inconsistent basal feeding behaviour on deviation detection efficacy, stimulating more consistent basal behaviour by improving pigs’ housing environment seems an interesting research direction. In addition, links between welfare and other behaviours that are more easily compromised in mildly welfare-reducing events, such as play or positive social interactions, deserve more research attention.

## Electronic supplementary material

Below is the link to the electronic supplementary material.

Supplementary Material 1

Supplementary Material 2

## Data Availability

The datasets generated and analysed during the current study are available in Mendeley Data, https://doi.org/10.17632/2mbw72m3g8.1. In addition, cleaned/processed versions of these data and analysis scripts are available from the authors upon request.
