# Supplementary material for: Co-occurrence of sudden feeding behaviour deviations and welfare issue onsets in growing-finishing pigs
Source: Porcine Health Manag. 2025 Aug 7;11:44. doi: 10.1186/s40813-025-00456-3 (PMC12333252; doi:10.1186/s40813-025-00456-3)
Supplement: Supplementary file 1 — Supplementary Material 1 [file 40813_2025_456_MOESM1_ESM.pdf]

Understanding sudden feeding behaviour deviations upon welfare issue onsets in growing-finishing pigs

Supplementary Methods

Jacinta D. Bus[1], Rudi M. de Mol[2], Laura E. Webb[1], Eddie A.M. Bokkers[1], Iris J.M.M. Boumans[1]

[1] *Animal Production Systems group, Wageningen University & Research, 6700AH Wageningen, the Netherlands*

[2] *Wageningen Livestock Research, 6708WD Wageningen, the Netherlands*

Corresponding author: Jacinta D. Bus. Email: jacintabus@outlook.com

1. Quantitative data description

Supplementary Table S1 Quantifications of the welfare issue scores observed in this study, by pig observation day (i.e. before defining onsets). 'All' provides the total number of pig observation days and, for each welfare issue, the number of observation days with a score higher than 0. 'Pig round', 'Month' and 'Score' provide the same information, but split per round of pigs (there were 26 observation days in rounds 1 and 2, 27 in round 3, and 24 in round 4), month of the growing-finishing phase (month 1: d1-d30, month 2: d31-60, month 3: ≥ d61), or score level of the welfare issue (ranged from 0-4, an empty slot means the score did not apply for that welfare issue), respectively. 'Threshold' provides the threshold value from above which (≥) a welfare issue was considered 'severe' and was hence included as an issue (i.e. a score '1') in the binary models.

**Welfare issue**	**All**	**Pig round**				**Month**			**Score**					**Threshold**
		1	*2*	*3*	*4*	*1*	*2*	*3*	*0*	*1*	*2*	*3*	*4*	≥
Total obs. days	*10,749*	*2,664*	*2,670*	*2,856*	*2,559*	*3,595*	*3,449*	*3,705*	*10,749*	*10,749*	*10,749*	*10,749*	*10,749*	
Blue ear disease	1	0	0	0	1	0	1	0	10,748	1				1
Bursitis	36	9	4	8	15	18	11	7	10,711	33	3			1
Conjunctivitis	204		50	58	96	89	62	53	7,882	199	5			1

Welfare issue	All	Pig round				Month			Score					Threshold
		1	*2*	*3*	*4*	*1*	*2*	*3*	*0*	*1*	*2*	*3*	*4*	≥
Coughing[1]	218	22	20	0	176	11	52	155	10,522	218				
Ear base damage	810	311	68	172	259	445	206	159	9,939	625	141	40	4	2
Ear tip damage	1,392	104	295	729	264	197	400	795	9,358	1,289	82	5	16	2
Flank damage	395	25	88	50	232	197	118	80	10,353	334	61			1
Heat stress[2]	1,945	0	0	1,945	0	0	330	1,615	26,454	1,945				
Hernia	4	0	3	1	0	3	1	0	10,743	4				1
Lameness	431	65	81	75	210	78	104	249	10,319	339	84	8		2
Lesions (front)	6,220	991	1,510	1,856	1,863	2,393	1,969	1,858	4,529	5,981	230	9		2
Lesions (middle)	3,508	407	846	839	1,416	1,580	1,076	852	7,240	3,378	127	3		2
Lesions (rear)	3,474	361	1,113	968	1,032	1,243	1,037	1,194	7,269	3,378	96			2
Low body condition	13	0	13	0	0	0	4	9	10,737	13				1
Pumping	3	1	0	0	2	1	0	2	10,747	3				1
Rectal prolapse	70	43	13	0	14	3	22	45	10,680	69	1			1
Shivering	13	12	0	0	1	0	2	11	10,737	13				1
Tail damage	2,369	701	570	689	409	669	742	958	8,296	1,983	272	43	71	2

[1] The number of coughs per pen on an observation day was (mean ± sd) 1.1 ± 0.1, ranging from 0 – 48. At farm level, the number of coughs on an observation day was 11 ± 2.4, ranging from 0 – 129. Most coughs occurred in pig round 4 (round 1: 5.7 ± 2.2 (0 – 45); round 2: 2.4 ± 1.5 (0 – 35); round 3: 0.5 ± 0.2 (0 – 5); round 4: 37.8 ± 7.7 (0 – 129)) and later in the growing-finishing phase (month 1: 6.7 ± 1.8 (0 – 45); month 2: 7.5 ± 2.4 (0 – 54); month 3: 17.6 ± 5.9 (0 – 129)). In this table, coughing score '1' represents a pig day upon which there were ≥ 15 coughs within that pig's pen during the 5 min-observation period.

[2] Heat stress score '1' represents a day upon which the temperature humidity index (THI) ≥ 79 and ≥ 10% of pigs were panting. Unlike the other welfare issues, heat stress was determined for each pig day, not each observation day. The total number of pig days was 38,235, with 9,391, 9,479, 10,513 and 8,852 pig days in rounds 1 – 4, respectively, and with 12,966, 12,937, 12,332 pig days in months 1 – 3, respectively.

Supplementary Table S2 A quantitative summary of the onsets of welfare issues, which was what the alerts of the dynamic linear model were compared to. For each welfare issue, given are the total number of onsets, the total number of onsets for which sufficient feeding data were available to allow comparison to the alerts ("Included"), and for each pig and pen 1) how many unique pigs/pens had an onset of this issue; 2) how many onsets an individual pig/pen encountered (mean ± se), and 3) the range (min – max) of onsets per pig/pen. Coincidence quantifies for what percentage of onsets there was also a onset for at least one other welfare issue.

**Welfare issue**	**Total**	**Included**	**Pig**			**Pen**			**Coincidence**
	#	#	*Unique*	*mean ± se*	*range*	*Unique*	*mean ± se*	*range*	%
Any issue	1,632	1,525	409	3.7 ± 0.1	0 – 11	40	40.8 ± 2.8	8 – 78	
Blue ear disease	1	1	1	0.0 ± 0.0	0 – 1	1	0.0 ± 0.0	0 – 1	0.0
Bursitis	31	21	28	0.1 ± 0.0	0 – 3	18	0.8 ± 0.2	0 – 8	19.4
Conjunctivitis	178	166	101	0.4 ± 0.0	0 – 9	30	4.4 ± 0.6	0 – 16	11.2
Coughing	165	161	116	0.4 ± 0.0	0 – 2	11	4.1 ± 1.1	0 – 22	7.3
Ear base damage	120	117	82	0.3 ± 0.0	0 – 5	23	3.0 ± 0.8	0 – 22	30.0
Ear tip damage	77	76	54	0.2 ± 0.0	0 – 4	24	1.9 ± 0.5	0 – 19	26.0
Flank damage	263	247	180	0.6 ± 0.0	0 – 5	38	6.6 ± 0.9	0 – 25	15.2
Heat stress	512	467	110	1.2 ± 0.1	0 – 5	10	12.8 ± 3.6	0 – 55	0.0
Hernia	3	3	2	0.0 ± 0.0	0 – 2	2	0.1 ± 0.1	0 – 2	0.0
Lameness	76	71	56	0.2 ± 0.0	0 – 5	28	1.9 ± 0.3	0 – 8	17.1
Lesions (front)	202	194	171	0.5 ± 0.0	0 – 4	35	5.0 ± 0.6	0 – 12	51.0
Lesions (middle)	119	111	103	0.3 ± 0.0	0 – 3	30	3.0 ± 0.5	0 – 10	70.6
Lesions (rear)	90	87	79	0.2 ± 0.0	0 – 4	30	2.2 ± 0.4	0 – 11	48.9
Low body condition	2	2	1	0.0 ± 0.0	0 – 2	1	0.0 ± 0.0	0 – 2	0.0
Pumping	3	3	3	0.0 ± 0.0	0 – 1	3	0.1 ± 0.0	0 – 1	0.0
Rectal prolapse	43	39	23	0.1 ± 0.0	0 – 4	15	1.1 ± 0.3	0 – 8	23.3
Shivering	10	10	5	0.0 ± 0.0	0 – 3	4	0.2 ± 0.1	0 – 3	20.0
Tail damage	217	196	160	0.5 ± 0.0	0 – 4	39	5.4 ± 0.8	0 – 26	14.3

Supplementary Table S3 For each of the four feeding strategy dimensions, the number of welfare issue bouts (T) that occurred among the pigs with a specific feeding strategy, and the number of unique pigs (U) affected. For comparison, the total number of onsets and the total number of affected unique pigs are also given (Total).

**Welfare issue**	**Total**		**Nibbling/Meal eating**						**Fast/Slow eating**						**Day/Day-night eating**						**Consistent/Inconsistent eating**					
			**Nibbler**		**Inter.**		**Meal eater**		**Fast**		**Inter.**		**Slow**		**Day**		**Inter.**		**Day/night**		**Cons.**		**Inter.**		**Incons.**	
	T	*U*	*T*	*U*	*T*	*U*	*T*	*U*	*T*	*U*	*T*	*U*	*T*	*U*	*T*	*U*	*T*	*U*	*T*	*U*	*T*	*U*	*T*	*U*	*T*	*U*
Flank damage	263	180	86	59	96	59	78	59	87	62	85	54	88	61	81	55	83	56	96	66	86	54	88	63	86	60
Heat stress	512	110	175	36	171	37	165	36	167	36	173	37	171	36	172	36	177	37	162	36	175	36	172	37	164	36
Lameness	76	56	24	17	20	19	31	19	33	20	19	17	23	18	25	15	27	20	23	20	34	25	20	15	21	15
Tail damage	217	160	71	55	54	45	65	49	58	48	64	47	68	54	68	48	51	42	71	59	63	51	64	48	63	50

2. Extended description of '2.3.1.2 Predicting on-line (day-by-day)'

A dynamic linear model and Kalman filter were used to estimate the values of the variables in Equations 2.1-2.5 on a day-by-day basis, as previously described in [1–3]. This process estimated the 'state' of the system (i.e. the feeding behaviour component) at any given time, including the set of variables that would be used to predict the coming state. The Kalman filter used an observation equation to describe the relationship between the previous state of the system and the new observation (Equation S1.1), and a system equation to reflect that the state of the system could change across time (Equation S1.2).

$$y_t = C_t \cdot x_{t-1} + v_t \,, \quad v_t \sim N(0, V_t) \qquad (S1.1)$$

$$x_t = A_t \cdot x_{t-1} + w_t \,, \quad w_t \sim N(0, W_t) \qquad (S1.2)$$

in which: y_t = observation vector at time t; C_t = the design matrix at time t, which describes the relation between the observation and the state of the system; x_t = state at time t; v_t = observational error at time t, normally distributed with zero average and variance V_t; A_t = the system matrix at time t, which describes the relation between the previous and the current state; w_t = system error at time t, normally distributed with zero average and variance W_t.

The observation (S1.1) and system (S1.2) equations could be equated to the extended quadratic trend model (Equations 2.1-5 in the main manuscript) by defining $y_t = Y_t$; $x_t = (\mu_t\ \alpha_t\ \beta_t\ \gamma_t)^T$; $C_t = (1\ 0\ 0\ \overline{Y}_t)$; and A_t = (1,1,1,0; 0,1,1,0; 0,0,1,0; 0,0,0,1).

The prediction procedure using these equations consisted of two stages: a prediction stage and an updating stage. In the prediction stage, the estimate of the coming state was based on the previous state:

$$\hat{x}_{t|t-1} = A_t \cdot \hat{x}_{t-1} \qquad (S1.3)$$

along with the variance-covariance matrix:

$$P_{t|t-1} = A_t \cdot P_{t-1} \cdot A_t^T + W_t \qquad (S1.4)$$

in which: $\hat{x}_{t|t-1}$ = estimate of state x at time t using all information up to time t-1; $\hat{x}_{t-1}$ = estimate of state x at time t-1 using all information up to time t-1; $P_{t|t-1}$ = estimate of the variance-covariance matrix P at time t using all information up to time t-1; P_{t-1} = estimate of the variance-covariance matrix P at time t-1 using all formation up to time t-1.

In the updating stage, the obtained estimate was updated with the real observation y_t and the estimation error (e_t, Equation S1.5) and its variance (E_t, Equation S1.6) were calculated:

$$e_t = y_t - C_t \cdot \hat{x}_{t|t-1} \qquad (S1.5)$$

$$E_t = C_t \cdot P_{t|t-1} \cdot C_t^T + V_t \qquad (S1.6)$$

With this estimation error, an improved estimate of the state could be made:

$$\hat{x}_t = \hat{x}_{t|t-1} + K_t \cdot e_t \qquad (S1.7)$$

with variance-covariance matrix:

$$P_t = P_{t|t-1} - K_t \cdot C_t \cdot P_{t|t-1} \qquad (S1.8)$$

In these equations, the matrix K_t, referred to as the Kalman gain, represented the influence of the error at time t on the state estimate. From Equations S1.5 and S1.7, it can be derived that K_t also represents the influence of the current observation on the state estimate, namely:

$$\hat{x}_t = K_t \cdot y_t + (I - K_t \cdot C_t) \cdot \hat{x}_{t|t-1} \qquad (S1.9)$$

in which I is the identity matrix. K_t itself was defined as:

$$K_t = P_{t|t-1} \cdot C_t^T \cdot [C_t \cdot P_{t|t-1} \cdot C_t^T + V_t]^{-1} \qquad (S1.10)$$

The estimates obtained during this process could be used to create alerts (see manuscript section 2.3.1.3) and to predict the system's state (i.e. pig behaviour) for the next time step t+1.

References

1. De Bruijn BGC, De Mol RM, Hogewerf PH, Van der Fels JB (2023) A correlated-variables model for monitoring individual growing-finishing pig's behavior by RFID registrations. Smart Agricultural Technology. https://doi.org/10.1016/j.atech.2023.100189
2. De Mol RM, André G, Bleumer EJB, Van der Werf JTN, De Haas Y, Van Reenen CG (2013) Applicability of day-to-day variation in behavior for the automated detection of lameness in dairy cows. Journal of Dairy Science 96:3703–3712
3. De Mol RM (2000) Automated detection of oestrus and mastitis in dairy cows (PhD thesis). 1–178
