# Supplementary material for: Co-occurrence of sudden feeding behaviour deviations and welfare issue onsets in growing-finishing pigs
Source: Porcine Health Manag. 2025 Aug 7;11:44. doi: 10.1186/s40813-025-00456-3 (PMC12333252; doi:10.1186/s40813-025-00456-3)
Supplement: Supplementary file 2 — Supplementary Material 2 [file 40813_2025_456_MOESM2_ESM.pdf]

Understanding sudden feeding behaviour deviations upon welfare issue onsets in growing-finishing pigs

Supplementary Results

Jacinta D. Bus[1], Rudi M. de Mol[2], Laura E. Webb[1], Eddie A.M. Bokkers[1], Iris J.M.M. Boumans[1]

[1] *Animal Production Systems group, Wageningen University & Research, 6700AH Wageningen, the Netherlands*

[2] *Wageningen Livestock Research, 6708WD Wageningen, the Netherlands*

Corresponding author: Jacinta D. Bus. Email: jacintabus@outlook.com

1. Sensitivity changes in pigs with specific feeding strategies

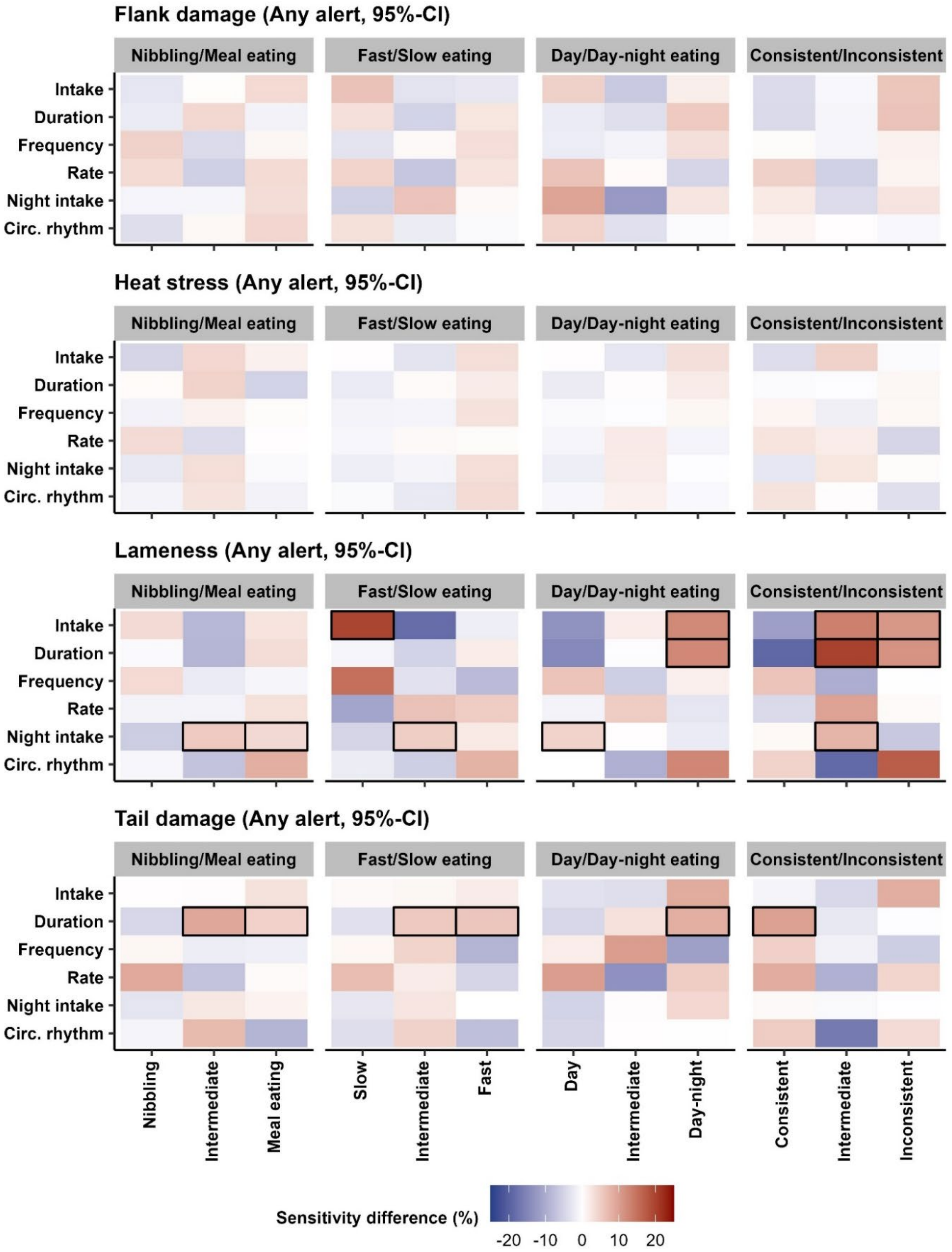

Supplementary Figure S1 For each subgroup of pigs with a specific feeding strategy, cell colour indicates how much better or worse alerts indicative of sudden changes in feeding patterns (any alert at a 95%-confidence interval (CI)) corresponded with each of four example welfare issues compared to for all pigs simultaneously. Sensitivity difference is given in absolute %, for example -10% may represent a change from 40% to 30%. If subgroup sensitivity exceeded 50%, the cell is outlined in black.

2. Exploration on individual variation in alert frequency

One of this study's findings were that the alert frequency was highly variable between pigs, ranging from 1.1% to 22.2% for both positive and negative alerts across feeding components (Table 1). As mentioned in the discussion, our main hypotheses to explain this result were that it would relate to pigs' feeding strategies, frequency of health issue onsets or pig dominance rank. For example, pigs with more health issue onsets may also have a higher number of alerts, as may pigs with very consistent or very inconsistent feeding strategies as opposed to intermediates. As data on feeding strategies and health issues were available, we performed a first exploration to test some of these hypotheses. This part of the supplementary methods presents this exploration in text and visualisations. We first explore basal information on alert frequency distributions and overlap between feeding components, and subsequently compare distributions across pigs with different feeding strategies and welfare issue onset frequencies.

The first thing we looked into was how the alert frequencies were distributed. Were the very high or low frequencies outliers? Did distributions approximate normal or were they skewed? Supplementary Figure S2 presents the distributions. For the majority of feeding components, alert frequency distributions approximated normal. The minima reported in Table 1 seemed common occurrences, while the maxima seemed outliers. Without outliers, the maxima seem to be around 12% of days. An exception to the normal

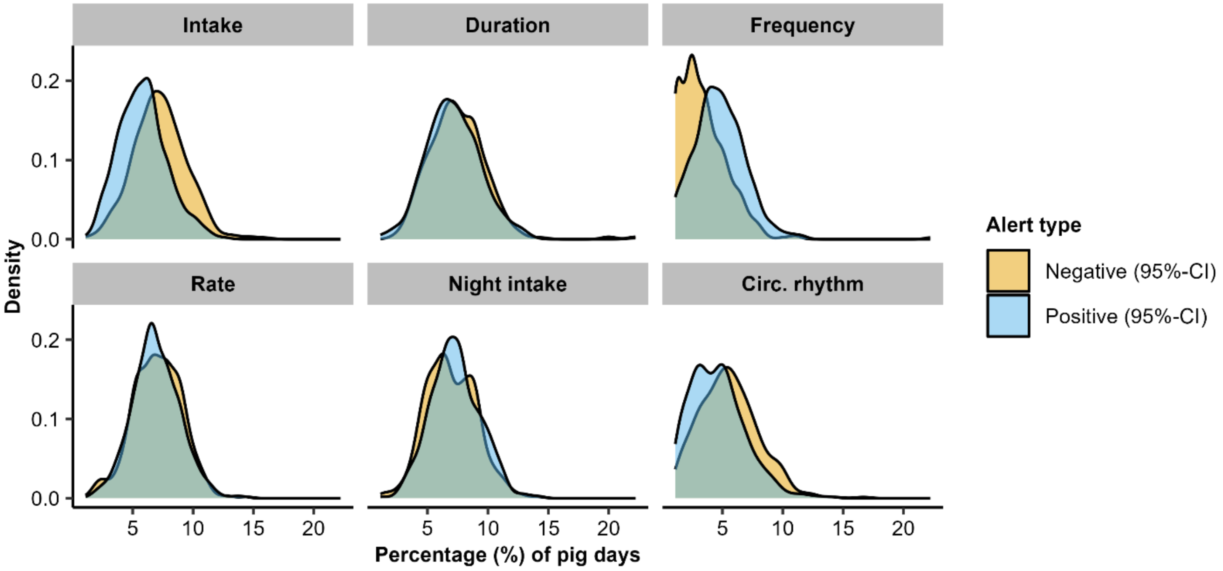

Supplementary Figure S2. Density plots of on how many days individual pigs had an alert, indicative of a behavioural deviation. Distribution are shown separately for each feeding component and for positive or negative alerts, always at a 95%-confidence interval (CI).

distributions was seen for negative alerts in feeding frequency, which had a higher density of low alert frequencies with a tail towards high frequencies. These distributions are onwards used to better interpret the figures on feeding strategies and welfare issue onsets.

Our second basal exploration was to check whether the individual low and high alert frequencies were consistent across feeding components. If so, that would suggest the presence of individuals that have few or many detectable behavioural deviations, regardless of which component of feeding behaviour is quantified. Supplementary Figure S3 presents a scatter plot of the alert frequency of individual pigs across feeding components. If pigs were consistent in deviation frequency across component, we would have expected high correlations and points nearing a linear line. This was, however, not observed; all plots contained a cloud of points with no clear pattern and Pearson correlation coefficients (obtained with the *ggpairs()* function of the *GGally* package [1]) were always lower than 0.3. Therefore, these results suggest that pigs could have variable alert frequencies across feeding components, and that this does not reflect an individual characteristic.

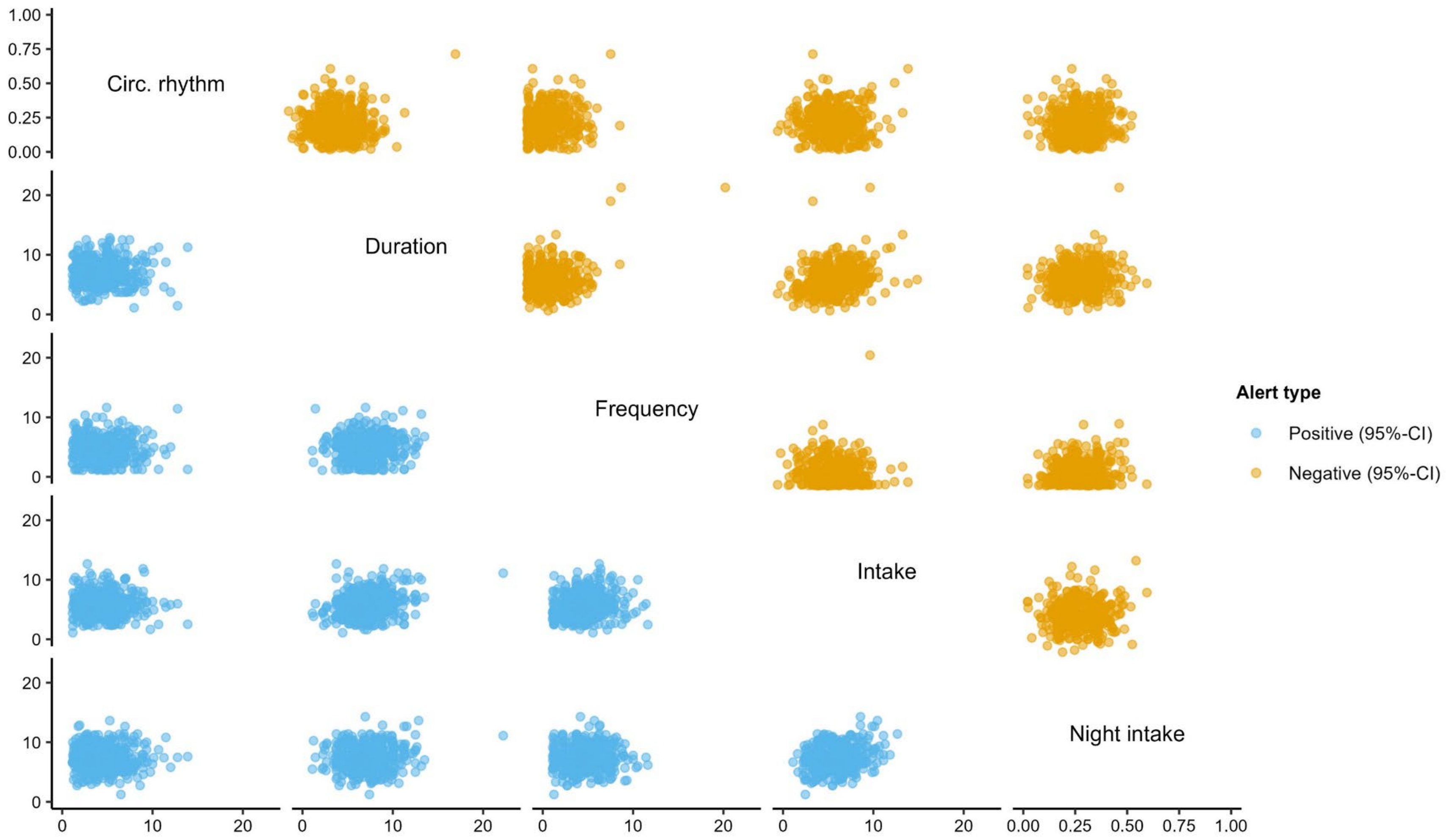

Supplementary Figure S3. Scatter plots comparing the percentage of days with positive or negative alerts (at 95%-confidence interval (CI)), indicative of behavioural deviations, between feeding components, at individual pig level.

Alert frequency could be lower or higher for pigs with specific feeding strategies. For example, pigs with very consistent or very inconsistent feeding behaviour from day-to-day may have a higher alert frequency than pigs with intermediate consistency, because from consistent patterns smaller deviations can be detected while very inconsistent patterns may contain more deviations. Supplementary Figure S4 shows the alert frequency distributions across groups of pigs with different feeding strategies. Applied feeding strategy groups were the same as for Section 3.3 and Figure 5 in the main manuscript. Distributions were very similar across feeding strategies, suggesting that feeding strategies are not a strong contributor to how many deviations are detected in individual pig behaviour. An exception is a lower frequency of alerts in feeding frequency for meal-eating pigs compared to nibblers and intermediates. This corresponds with the lower frequency alerts in feeding frequency overall that was seen in Supplementary Figure S2. Although this finding may be biologically-relevant, it could also reflect an impossibility of pigs with a low feeding frequency to further reduce the number of meals, making it an expected statistical artefact.

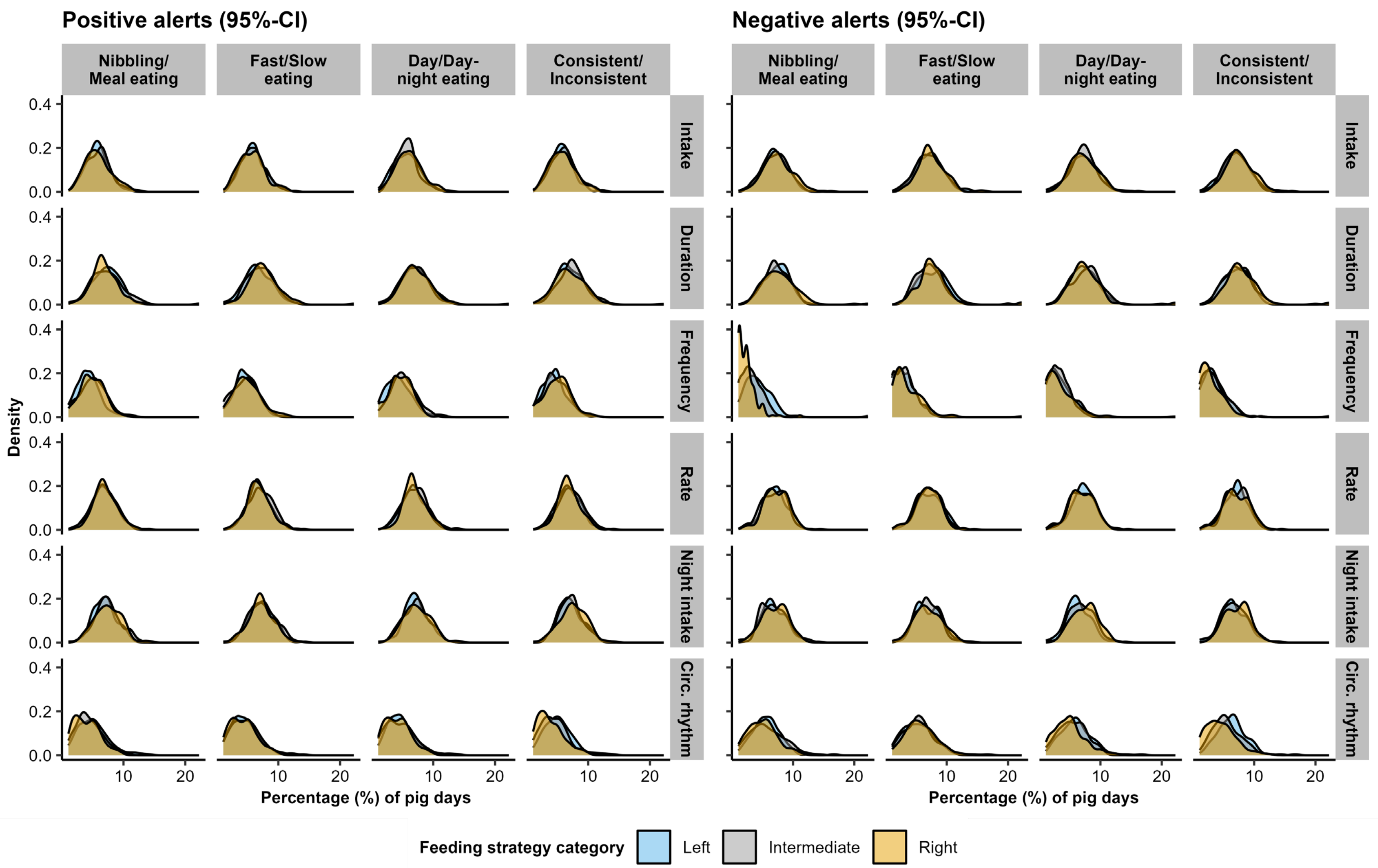

Supplementary Figure S4. Density plots of the percentage of days with alerts, indicative of deviations in feeding behaviour components, across pigs with different feeding strategies. Category 'left' refers to nibblers, slow eaters, day eaters and consistent eaters; category 'right' to meal eaters, fast eaters, day-night eaters and inconsistent eaters; and category 'intermediate' to those in between.

Finally, we compared whether a higher frequency of alerts corresponded with a higher frequency of health issue onsets. For each pig, the percentage of days with a health issue onset was calculated, using the same data as for the sensitivity calculations in the main manuscript. The resulting scatter plots are shown in Supplementary Figure S5. If the frequency of alerts and health issue onsets are related, we would expect the points to lie close to a linear line. This was, however, not the case, as all plots contain a cloud of points without any identifiable pattern, and all Pearson correlation coefficients (obtained with the *ggpairs()* function of the *GGally* package [1]) were smaller than 0.3. This suggests that there is no clear relationship between the frequency in detected behavioural deviations and the frequency of welfare issue onsets.

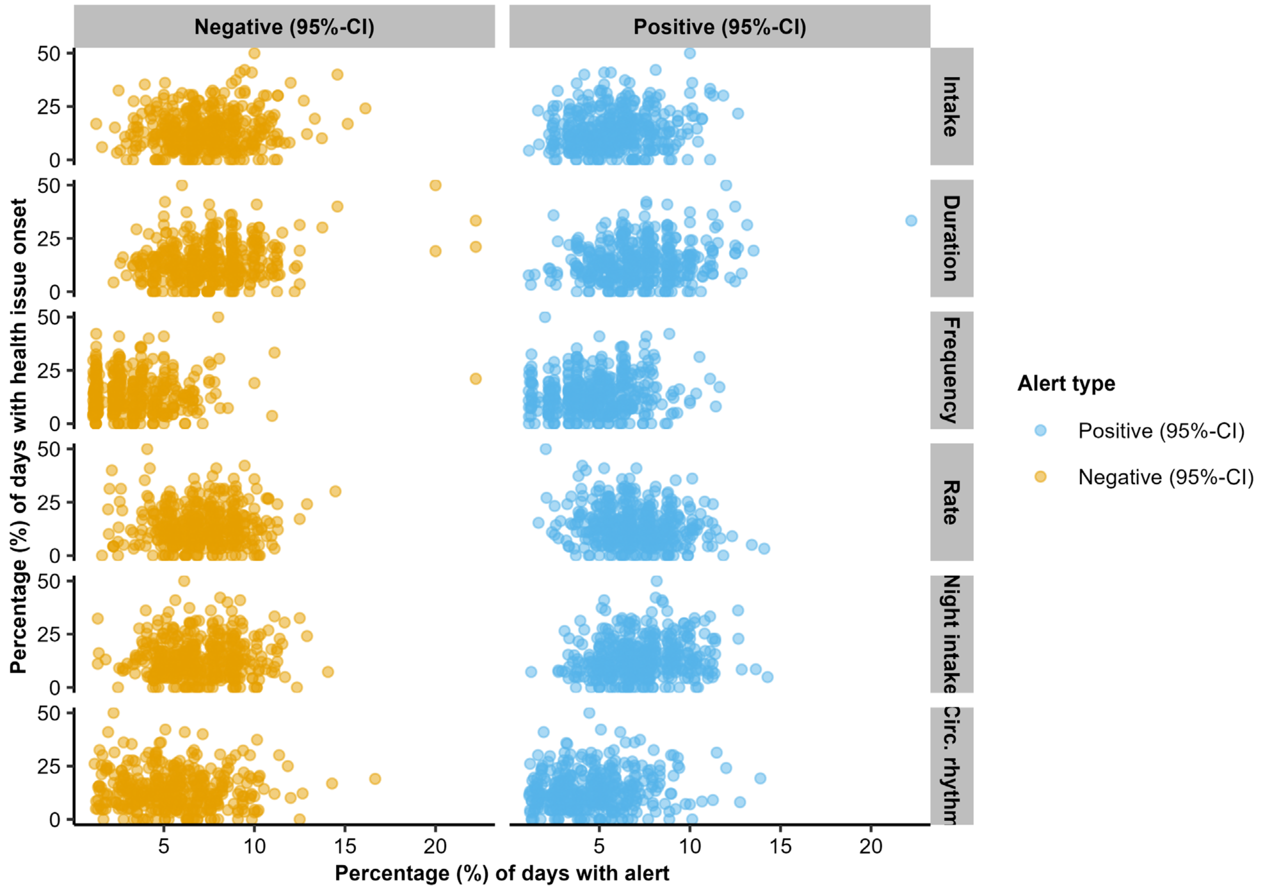

Supplementary Figure S5. Scatter plots of the percentage of days with alerts (all, positive or negative, 95%-confidence interval (CI)), indicative of deviations in feeding behaviour components, against the percentage of days with a new health issue onset, at individual pig level.

Overall, these explorations suggest that there is little value in further studying influences of feeding strategies and welfare issue onsets on individual alert frequency. In addition, the normal distributions and lack of overlap in alert frequency across feeding components suggests that the large range in individual alert frequencies may simply represent a statistical artefact. Nevertheless, there may be other biologically-relevant explanations for this large variation, such as pig dominance, which warrant further study.

References

1. Schloerke B, Cook D, Larmarange J, Briatte F, Marbach M, Thoen E, Elberg A, Crowley J (2024) GGally: Extension to 'ggplot2', https://ggobi.github.io/ggally/.
